# Traditional Chinese medicine in non-muscle-invasive bladder cancer: a scoping review of syndrome characteristics and critical synthesis of pharmacological and mechanistic evidence

**DOI:** 10.3389/fphar.2026.1921622

**Published:** 2026-09-22

**Authors:** Xi-Zhe Zhang, Jiong-Ao Xiang, Li-chang Yang, Yao-Dong Li, Xia Zheng, Jun Qian

**Affiliations:** 1 Department of Oncology, Jiangsu Province Hospital of Chinese Medicine, The Affiliated Hospital of Nanjing University of Chinese Medicine, Nanjing, Jiangsu, China; 2 Faculty of Chinese Medicine, Macau University of Science and Technology, Macao, China; 3 Beijing Friendship Hospital, Capital Medical University, Beijing, China; 4 Medical Affairs Department, The Fourth People’s Hospital of Shunde, Foshan (Wu Zhongpei Memorial Hospital of Shunde, Foshan), Foshan, Guangdong, China

**Keywords:** non-muscle-invasive bladder cancer, pharmacological evidence, scoping review, syndrome element, syndrome pattern, traditional Chinese medicine

## Abstract

Non-muscle-invasive bladder cancer (NMIBC) is characterized by frequent recurrence and prolonged surveillance. Traditional Chinese medicine (TCM) has been investigated as an adjunct to standard NMIBC management, but its syndrome characteristics and pharmacological evidence remain heterogeneous. This review combined a scoping review of TCM syndrome-related evidence with a critical narrative synthesis of pharmacological and mechanistic studies. The scoping review included 59 sources and identified 155 syndrome-element occurrences. Deficiency (23.9%), dampness (21.3%), heat (20.6%), stasis (16.8%), and toxin (14.8%) were most frequently reported. Among 59 composite-pattern occurrences, Dampness–Heat (30.5%) and Stasis–Toxin (18.6%) predominated. These results reflect literature-level reporting patterns rather than patient-level prevalence. Mechanistic evidence involved tumor-cell survival and death, inflammatory and immune regulation, angiogenesis, invasion, migration, extracellular-matrix remodeling, and tumor-cell reimplantation. However, much of this evidence derives from broader bladder cancer experimental models or predictive analyses, and direct evidence for reducing NMIBC recurrence or progression remains limited. Overall, TCM syndrome characteristics in NMIBC can be described systematically, but mechanistic findings should be interpreted according to their disease context and evidence relevance. Future studies should emphasize standardized syndrome reporting, reproducible intervention characterization, NMIBC-relevant models, and clinically meaningful recurrence-related outcomes.

## 1 Introduction

Bladder cancer is one of the most common malignancies of the urinary system, and its global disease burden continues to increase (Bray et al., 2018; Dyrskjøt et al., 2023). Non-muscle-invasive bladder cancer (NMIBC), including stage Ta, stage T1, and carcinoma *in situ*, accounts for approximately 75%–85% of newly diagnosed bladder cancer cases (Charlton et al., 2014; Sanli et al., 2017; Dobruch and Oszczudłowski, 2021). Although the overall prognosis of NMIBC is relatively favorable compared with muscle-invasive disease, its high recurrence rate, prolonged disease course, long-term surveillance burden, and heterogeneous risk of progression make it a major challenge in bladder cancer management.

The standard treatment for NMIBC consists of transurethral resection of bladder tumor (TURBT), followed by risk-adapted intravesical therapy. Intravesical *bacillus* Calmette–Guérin (BCG) or chemotherapeutic agents are commonly used to reduce postoperative recurrence (Wang et al., 2019; Sylvester et al., 2021). Nevertheless, recurrence and progression remain clinically significant. The 5-year recurrence rate may reach 31%–78% across different risk groups, whereas the 5-year progression rate ranges from 1% to 45% (Sylvester et al., 2006). In addition, responses to BCG vary substantially among individuals, and some patients develop BCG-unresponsive disease or recurrence after treatment. Repeated intravesical therapy may also cause adverse effects, including urinary frequency, urgency, dysuria, hematuria, and systemic discomfort, thereby affecting quality of life and treatment adherence (Cambier et al., 2016; Marttila et al., 2016; Tran et al., 2021; Lidagoster et al., 2024). Therefore, adjunctive strategies that can relieve symptoms, improve treatment tolerance, and support long-term management within the standard treatment framework remain clinically relevant.

Traditional Chinese medicine (TCM) has been investigated as an adjunctive approach in the management of NMIBC. Within TCM practice, NMIBC has been described using syndrome concepts such as healthy-qi deficiency, dampness–heat, blood stasis, and toxin-related patterns, with corresponding therapeutic principles including reinforcement of healthy qi, clearance of heat and dampness, activation of blood and resolution of stasis, and clearance of toxin or pathogenic accumulation (Yu et al., 2016; Li et al., 2017; Qiu et al., 2022). These syndrome concepts represent traditional diagnostic and therapeutic constructs and should not be interpreted as direct biological equivalents of specific molecular pathways. Clinical studies have investigated TCM interventions alongside standard postoperative or intravesical therapy, particularly in relation to treatment-related adverse effects, lower urinary tract symptoms, inflammatory or immune indices, and quality of life (Zhang et al., 2020; Wu et al., 2022; Ren et al., 2024). Separately, individual TCM formulae, preparations, plant-derived metabolites, and other defined natural products have been investigated in distinct clinical, experimental, or predictive studies for effects on tumor-cell survival, inflammatory and immune regulation, angiogenesis, invasion, migration, and related biological processes. The relevance and strength of these findings vary substantially across interventions, experimental systems, and disease contexts, and they should not be interpreted as demonstrating a general or unified pharmacological effect of TCM as a therapeutic class.

However, the available evidence remains heterogeneous at both the syndrome and pharmacological levels. Syndrome patterns and pathogenesis have been described using variable terminology, including “dampness–heat pouring downward,” “stasis–toxin interconnection,” and “spleen–kidney deficiency,” which limits comparison and synthesis across studies. Pharmacological evidence is similarly fragmented across different formulae, preparations, isolated metabolites or natural products, experimental systems, and disease contexts. In addition, the composition, preparation, dosage, botanical authentication, product traceability, and chemical characterization of multi-botanical-drug interventions are not consistently reported. Mechanistic findings derived from conventional bladder cancer cell models, animal experiments, isolated metabolites or other defined natural products, network pharmacology, molecular docking, or other predictive approaches cannot be assumed to represent the pharmacological effects of clinically used formulae or to translate directly to NMIBC recurrence and progression. These limitations require explicit separation of traditional syndrome evidence from pharmacological and mechanistic evidence and careful consideration of the experimental and clinical context of individual studies.

Accordingly, this review comprised two complementary evidence-synthesis components: a scoping review of TCM syndrome-related evidence in NMIBC and a critical narrative synthesis of pharmacological and mechanistic evidence. The scoping-review component was used to systematically identify, standardize, and deconstruct TCM syndrome patterns reported in NMIBC-related literature, whereas the pharmacological and mechanistic component critically evaluated evidence concerning specific formulae, preparations, plant-derived metabolites, and other defined natural products in NMIBC-specific and broader bladder cancer contexts. By separating syndrome-level evidence from pharmacological and mechanistic evidence and by explicitly considering study design, disease relevance, intervention characterization, and experimental context, this review aims to provide a more transparent and structured assessment of the current evidence for adjunctive TCM within biomedical NMIBC management and to identify priorities for future translational research.

## 2 Methods

### 2.1 Overall review design

This review comprised two complementary evidence-synthesis components: (1) a scoping review of TCM syndrome-related evidence in NMIBC and (2) a critical narrative synthesis of pharmacological and mechanistic evidence. The scoping-review component was conducted and reported with reference to the PRISMA Extension for Scoping Reviews (PRISMA-ScR).

### 2.2 Scoping review of TCM syndrome characteristics in NMIBC

The scoping-review component was used to characterize the terminology, standardization, and literature-level distribution of TCM syndrome patterns and syndrome elements in NMIBC.

#### 2.2.1 Eligibility criteria

Sources were eligible if they (1) included patients with NMIBC, or an explicitly identifiable NMIBC subgroup within a broader bladder cancer population; (2) reported extractable TCM syndrome-related information; and (3) allowed the reported syndrome information to be attributed to the eligible NMIBC population or subgroup.

Mixed-stage bladder cancer studies were eligible only when the NMIBC subgroup was explicitly identifiable and the reported syndrome information was directly applicable to that subgroup. Sources were excluded if they provided no extractable TCM syndrome information, contained insufficient information for predefined data charting, did not include an eligible or confirmable NMIBC population or NMIBC-specific syndrome data, or represented overlapping reports of the same study.

#### 2.2.2 Information sources and search strategy

CNKI, Wanfang Data, PubMed, and Web of Science Core Collection were searched for publications from 1 January 2000 to 31 December 2025, with the final search conducted on 5 August 2026. No language or publication-type restrictions were applied at the search stage.

Search strategies combined terms for bladder cancer/NMIBC with terms for traditional Chinese medicine. Database-specific controlled vocabulary, free-text terms, and search fields were used as appropriate. Syndrome-related eligibility was determined during screening rather than by restricting the search to syndrome-specific terms. Full electronic search strategies are provided in Supplementary Table S2.

#### 2.2.3 Source selection

Retrieved records were imported into Zotero and duplicate records were identified using Zotero’s duplicate-detection function. Potential duplicate records were manually reviewed on the basis of bibliographic information, including title, authors, publication year, journal, and DOI where available, before removal. The deduplicated records were then screened by title and abstract, followed by full-text eligibility assessment. Title/abstract screening, full-text assessment, and data extraction were independently performed by two researchers, with disagreements resolved through discussion. The complete selection process and reasons for full-text exclusion are shown in Figure 1.

**FIGURE 1:**
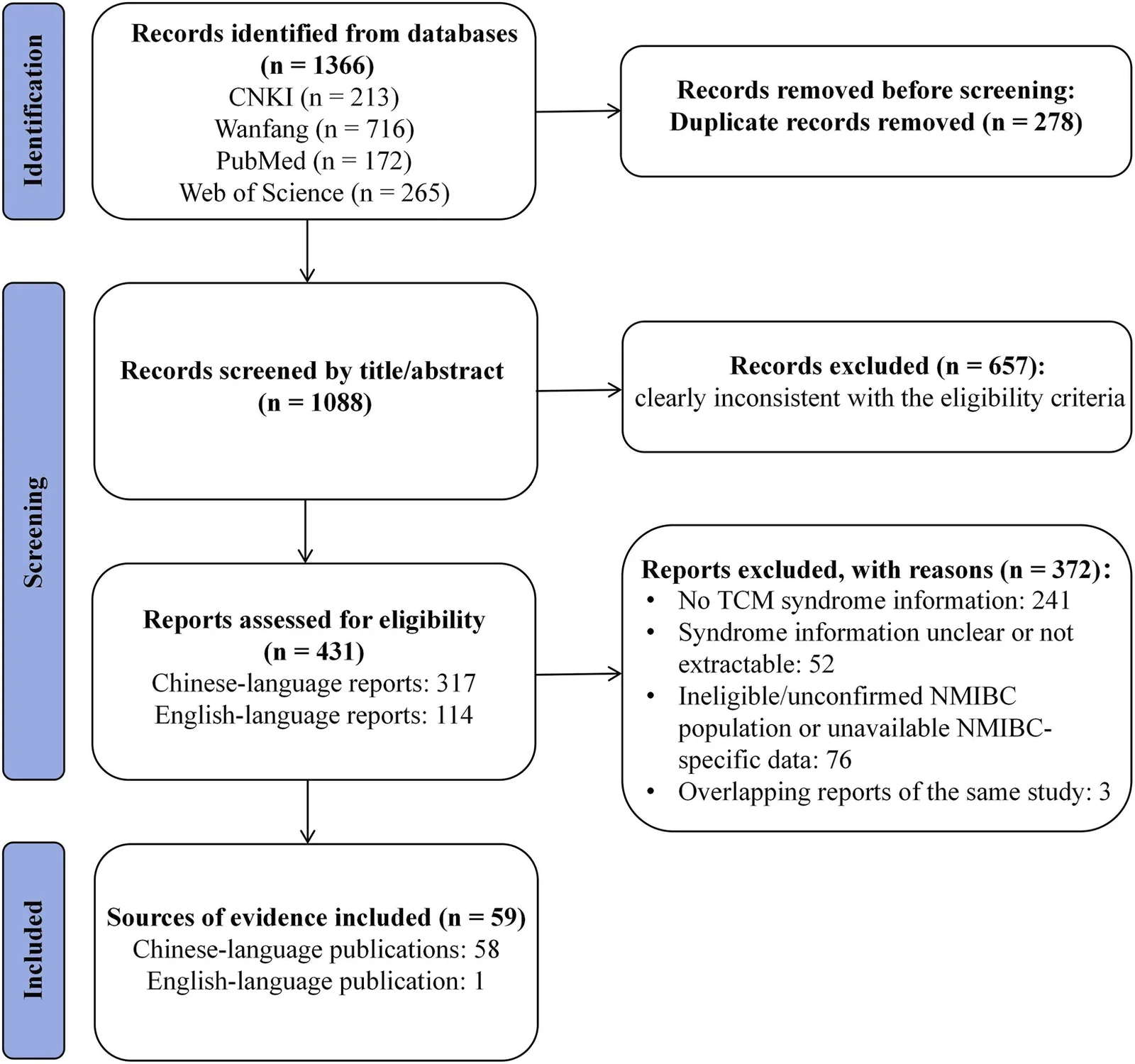
PRISMA-ScR flow diagram of the source selection process.

#### 2.2.4 Data charting and syndrome coding

A structured data-charting form was used to record source characteristics, clinical context, original syndrome terminology, syndrome-classification basis, standardized syndrome patterns, composite syndrome-element patterns, decomposed syndrome elements, and deficiency-related subtype descriptions (Supplementary Table S1).

Original syndrome terms were retained and standardized where terms had equivalent or closely related meanings. Composite patterns were preserved for composite-pattern analysis and were also decomposed into broader syndrome elements for single-element analysis. For example, dampness–heat-related patterns were decomposed into Dampness and Heat, whereas stasis–toxin-related patterns were decomposed into Stasis and Toxin.

Deficiency-related descriptions were grouped under the broad Deficiency domain in the primary analysis to maintain a consistent hierarchical level across syndrome elements. Original deficiency subtype descriptions were retained for separate sensitivity analysis.

Within each source, a given standardized syndrome element or composite pattern was counted no more than once, although one source could contribute to multiple distinct categories.

#### 2.2.5 Data synthesis

Syndrome characteristics were summarized descriptively using source-level occurrences. Frequencies were calculated as follows:

Syndrome-element frequency (%) = occurrences of a given syndrome element/total syndrome-element occurrences × 100%.

Composite syndrome-pattern frequency (%) = occurrences of a given composite pattern/total composite-pattern occurrences × 100%.

These frequencies represent literature-reported occurrence patterns rather than patient-level prevalence estimates.

Syndrome-element reporting was descriptively stratified by source type into patient-based empirical and experience-based sources (Supplementary Table S3). A sensitivity analysis additionally examined the distribution of original deficiency subtypes within the broad Deficiency domain (Supplementary Table S4). These analyses were descriptive, and no inferential between-group hypothesis testing was performed.

Because the scoping-review component was designed to map literature-reported syndrome terminology and occurrence patterns rather than to estimate treatment effects or patient-level prevalence, formal risk-of-bias scores were not used as eligibility criteria or as weights in the frequency calculations. Source type and syndrome-classification information were charted descriptively, and stratified and sensitivity analyses were used to examine the robustness of the reported syndrome profile.

### 2.3 Critical narrative synthesis of pharmacological and mechanistic evidence

The pharmacological and mechanistic component was conducted separately from the scoping review and was not included in the PRISMA-ScR flow or syndrome-frequency calculations. A targeted, literature-guided narrative approach was used, anchored to representative TCM formulae and preparations identified from the syndrome-related literature. Relevant studies were searched primarily in PubMed, Web of Science Core Collection, CNKI, and Wanfang Data, supplemented by citation tracking and targeted web-based searches where necessary. Searches combined the names of individual formulae, preparations, metabolites, or defined natural products with terms related to bladder cancer, urothelial carcinoma, NMIBC, pharmacology, and relevant biological mechanisms.

Formula- and preparation-level studies were included when the investigated intervention was clearly identifiable and the study reported clinical, experimental, or mechanistic findings relevant to NMIBC or bladder cancer. At the metabolite or defined-natural-product level, evidence was included only when the cited original study directly investigated the respective substance in a bladder cancer-related experimental or translational setting. Such findings were interpreted as evidence concerning the individual substance and were not used to infer the pharmacological effects of multi-botanical-drug formulae containing related botanical drugs. Studies confined to non-bladder malignancies were not used to support NMIBC- or bladder cancer-specific mechanistic claims. Computational or predictive analyses, including network pharmacology, molecular docking, bioinformatics, and predictive transcriptomics, were treated as hypothesis-generating evidence rather than equivalent to experimental or clinical validation.

The synthesis focused on tumor-cell proliferation and cell death, inflammatory and immune regulation, angiogenesis, invasion, migration, extracellular-matrix remodeling, and tumor-cell reimplantation. NMIBC-specific clinical and translational evidence was distinguished from evidence derived from broader bladder cancer cell or animal models.

For evidence stratification, individual studies were independently assessed by two reviewers using prespecified evidence-relevance strata: A, direct NMIBC clinical evidence; B, NMIBC-relevant translational evidence from postoperative, intravesical, orthotopic, reimplantation, or related models; C, experimental evidence from general bladder cancer models without specific NMIBC relevance; D, broader bladder cancer clinical or meta-analytic evidence without separately identifiable NMIBC data; and E, indirect or hypothesis-generating evidence derived from computational or predictive analyses. Multiple strata were assigned where applicable. Disagreements were resolved through discussion, with third-reviewer adjudication if required. These strata describe study design and disease-context relevance rather than formal levels of evidence quality.

In addition, reporting characteristics of formula- and preparation-level studies were descriptively assessed with reference to the Four Pillars of best practice in ethnopharmacology and the ConPhyMP recommendations (Heinrich et al., 2020; 2022), focusing on composition and dose/ratio reporting, preparation and administration, material source/authentication and product traceability, and phytochemical characterization or quality control (Supplementary Table S7). Taxonomic and source identification of medicinal materials used in representative formulae and preparations was additionally verified using MPNS, POWO, the Chinese Pharmacopoeia, and appropriate non-botanical taxonomic or source databases, as applicable (Supplementary Table S8).

## 3 Modern medical basis for NMIBC recurrence and progression

### 3.1 Clinical characteristics and recurrence risk of NMIBC

Non-muscle-invasive bladder cancer (NMIBC) refers to urothelial carcinoma that has not invaded the muscularis propria of the bladder, mainly including stage Ta, stage T1, and carcinoma *in situ* (CIS). Although the overall prognosis of NMIBC is better than that of muscle-invasive bladder cancer, its high recurrence rate, prolonged disease course, and substantial long-term management burden make it an important issue in bladder cancer care.

The risks of recurrence and progression in NMIBC are highly heterogeneous. Pathological stage, tumor grade, tumor size, multifocality, concomitant CIS, and prior recurrence history are all associated with long-term outcomes (Chang et al., 2016; Babjuk et al., 2022). In 2021, the European Association of Urology (EAU) updated its NMIBC risk stratification model by adding a very-high-risk group to the previous low-, intermediate-, and high-risk categories, thereby refining progression risk assessment (Sylvester et al., 2021). Previous studies have shown that the 5-year recurrence rate of NMIBC may reach 31%–78%, and some high-risk patients also have a substantial risk of progression (Tan et al., 2016). Frequent recurrence not only increases the burden of cystoscopic surveillance, repeat TURBT, and long-term follow-up, but also has a sustained impact on quality of life and treatment adherence. Therefore, recurrence control and long-term management remain central issues in the treatment of NMIBC. This clinical background provides the basis for further discussing biological mechanisms and adjunctive management strategies.

### 3.2 Standard treatment strategies and their limitations

At present, standard management of NMIBC is based on transurethral resection of bladder tumor (TURBT), followed by risk-adapted intravesical therapy and surveillance (Babjuk et al., 2019; Urologic Oncology Collaborative Group of Chinese Cancer Hospitals, 2025). For patients with high-risk disease, intravesical *bacillus* Calmette–Guérin (BCG) remains a major bladder-preserving treatment option (Holzbeierlein et al., 2024). Technical refinements, including *en bloc* TURBT and blue-light cystoscopy, have also been explored or incorporated into contemporary management to improve tumor detection and resection in selected settings (Daneshmand et al., 2018; Palakurthi et al., 2019; Zengin et al., 2022; Teoh et al., 2023; Necchi et al., 2024).

The therapeutic landscape of NMIBC is also evolving rapidly beyond conventional intravesical chemotherapy and BCG. For selected patients with BCG-unresponsive high-risk NMIBC, bladder-preserving treatment options now include systemic immune-checkpoint inhibition with pembrolizumab, intravesical gene therapy with nadofaragene firadenovec, and sustained intravesical gemcitabine delivery with the gemcitabine intravesical system (Inlexzo; formerly TAR-200) (Balar et al., 2021; Boorjian et al., 2021; Daneshmand et al., 2025). In BCG-naïve high-risk NMIBC, checkpoint-inhibitor combinations with BCG are also reshaping treatment strategies: the phase III CREST trial demonstrated improved event-free survival with sasanlimab plus BCG compared with BCG alone, and durvalumab plus BCG received regulatory approval in the United States in 2026 (Shore et al., 2025; Research, 2026). These developments provide an increasingly complex clinical context in which any adjunctive TCM strategy must be evaluated.

However, current treatment strategies still have several limitations. Some patients experience repeated recurrence after TURBT and require long-term cystoscopic surveillance, repeat TURBT, and intravesical therapy (Brausi et al., 2002). In addition, the efficacy of BCG varies considerably among individuals, and some patients develop recurrence or BCG-unresponsive disease after treatment (Jeong and Ku, 2023). Long-term and repeated intravesical therapy may also cause local irritative symptoms, such as urinary frequency, urgency, dysuria, and hematuria, which can adversely affect quality of life and treatment adherence (Nayak et al., 2021; Bourlotos et al., 2024; Gaylis et al., 2024). Therefore, it remains important to explore optimized adjunctive interventions within, rather than outside, the standard treatment framework to reduce recurrence risk and improve the quality of long-term management.

### 3.3 Recurrence-associated microenvironment and potential entry points for adjunctive TCM intervention

Postoperative recurrence of NMIBC is not determined by a single factor, but rather results from the combined effects of multiple processes, including residual lesions, intraoperative tumor-cell reimplantation, insufficient response to intravesical therapy, and persistent dysregulation of the local inflammatory and immune microenvironment (Pode et al., 1986; Hyacinthe et al., 1995; Brausi et al., 2002; Mariappan et al., 2010; Lidagoster et al., 2024). Recent studies have shown that recurrence and progression of NMIBC are associated not only with tumor-intrinsic factors, but also with microenvironmental changes, including chronic inflammation, altered immune-cell infiltration, angiogenesis, hypoxic responses, and stromal remodeling (Vermeulen et al., 2015; Fernando et al., 2017; Schneider et al., 2019; Liang et al., 2023; Kamitani et al., 2024). The marked interindividual variability in BCG efficacy further suggests that NMIBC is not characterized by a single immune phenotype; instead, local immune activation and immunosuppressive states may coexist over time (Miyake et al., 2017; Annels et al., 2020).

These biological characteristics provide a context for evaluating adjunctive TCM evidence but should not be interpreted as direct biological equivalents of TCM syndrome concepts. Clinically, TCM has primarily been investigated as an adjunct to standard NMIBC treatment for symptom management, treatment tolerance, and quality of life during the postoperative period and intravesical therapy. Pharmacological and mechanistic evidence concerning TCM formulae, preparations, plant-derived metabolites, and other defined natural products is evaluated separately in Section 5 according to disease context and evidence relevance. This distinction helps avoid overinterpreting traditional syndrome concepts as direct molecular or biological counterparts while allowing the clinical and mechanistic evidence to be considered within their respective evidentiary contexts.

## 4 Scoping review results and TCM syndrome characteristics in NMIBC

### 4.1 Source selection and characteristics

The database search identified 1,366 records, including 213 from CNKI, 716 from Wanfang Data, 172 from PubMed, and 265 from Web of Science Core Collection. After removal of 278 duplicates, 1,088 records underwent title and abstract screening, and 431 reports were assessed in full text. Of these, 372 were excluded because they provided no TCM syndrome information (n = 241), contained insufficiently clear or extractable syndrome information (n = 52), involved an ineligible or unconfirmed NMIBC population or lacked NMIBC-specific syndrome data (n = 76), or represented overlapping reports of the same study (n = 3). Ultimately, 59 sources were included in the scoping review (Figure 1).

Of the 59 included sources, 58 were Chinese-language publications and one was an English-language publication. Forty-two were journal articles and 17 were theses; 51 were classified as patient-based empirical sources and eight as experience-based sources. Detailed source characteristics and syndrome-coding information are provided in Supplementary Table S1.

### 4.2 Distribution of syndrome elements and composite syndrome patterns

Decomposition of the standardized syndrome patterns yielded 155 syndrome-element occurrences. Under the broad-domain coding scheme, Deficiency was the most frequently represented category (37/155, 23.9%), followed by dampness (33/155, 21.3%), heat (32/155, 20.6%), stasis (26/155, 16.8%), and toxin (23/155, 14.8%). Qi stagnation (3/155, 1.9%) and phlegm (1/155, 0.6%) were uncommon (Figure 2A).

**FIGURE 2:**
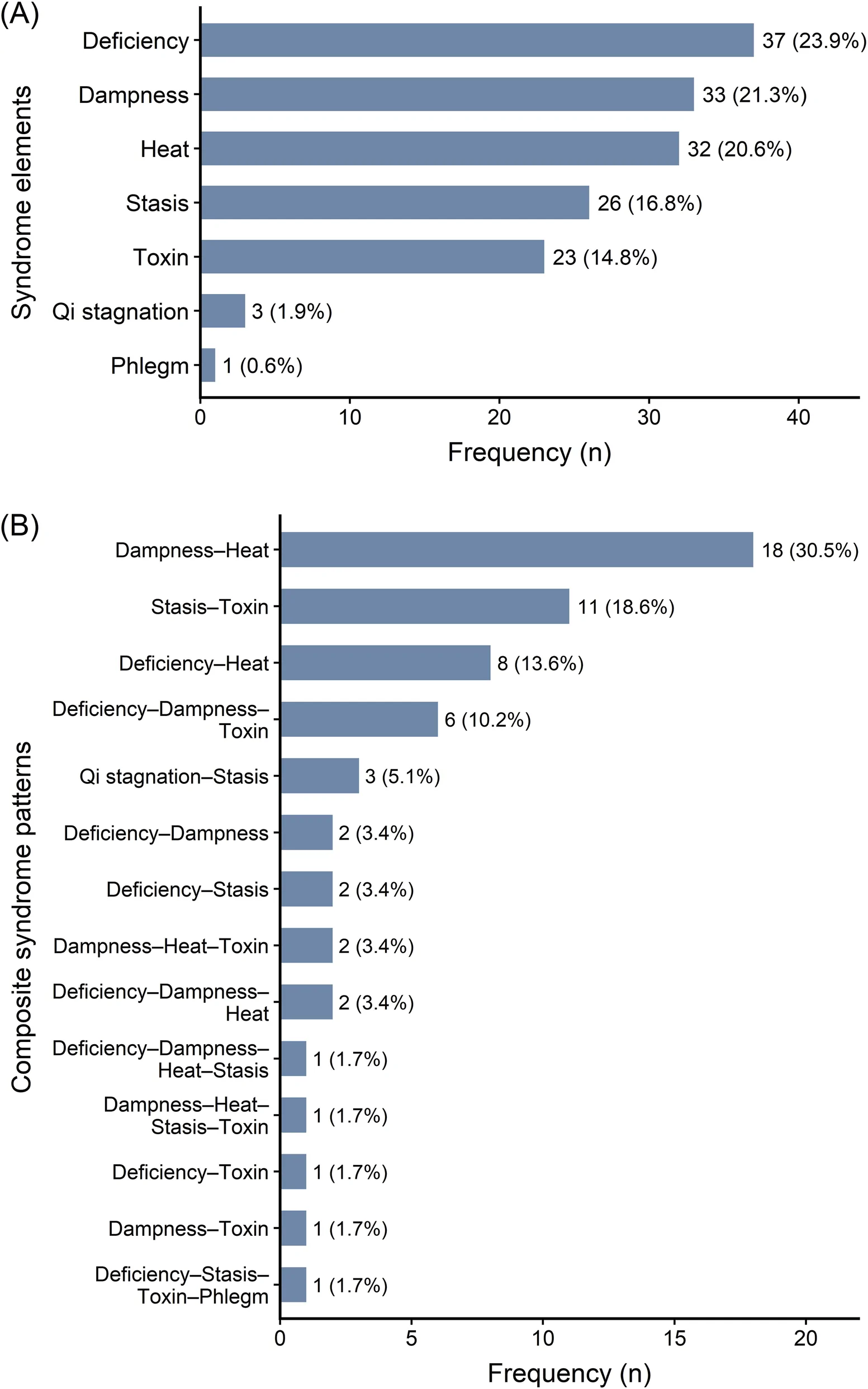
Distribution of traditional Chinese medicine syndrome characteristics among the included sources of evidence. Figure legend: **(A)** Frequencies of decomposed syndrome elements. Percentages were calculated as the number of occurrences of each syndrome element divided by the total number of syndrome-element occurrences (n = 155). **(B)** Frequencies of composite syndrome-element patterns. Percentages were calculated as the number of occurrences of each composite syndrome pattern divided by the total number of composite-pattern occurrences (n = 59). Individual sources could contribute more than one syndrome element or composite syndrome pattern; therefore, the frequencies represent literature-level occurrence patterns rather than patient-level prevalence.

Composite-pattern coding yielded 59 occurrences. Dampness–Heat was the most frequent combination (18/59, 30.5%), followed by Stasis–Toxin (11/59, 18.6%), Deficiency–Heat (8/59, 13.6%), and Deficiency–Dampness–Toxin (6/59, 10.2%). Qi stagnation–Stasis accounted for 5.1% (3/59), whereas Deficiency–Dampness, Deficiency–Stasis, Dampness–Heat–Toxin, and Deficiency–Dampness–Heat each accounted for 3.4% (2/59). The remaining composite patterns were individually reported once (1.7% each) (Figure 2B).

Overall, the literature-reported syndrome profile was dominated by deficiency, dampness, heat, stasis, and toxin, with Dampness–Heat and Stasis–Toxin representing the most frequently reported composite patterns.

### 4.3 Stratified and sensitivity analyses

The major syndrome-element profile was broadly consistent across source types (Supplementary Table S3). In patient-based empirical sources, deficiency (31/136, 22.8%), heat (29/136, 21.3%), and dampness (28/136, 20.6%) were most frequent; in experience-based sources, deficiency (6/19, 31.6%), dampness (5/19, 26.3%), and heat (3/19, 15.8%) predominated. Although the relative ordering differed, the same three major elements remained prominent in both strata.

Sensitivity analysis of the broad Deficiency domain identified 44 original deficiency-subtype occurrences across 37 sources (Supplementary Table S4). Qi deficiency (11/44, 25.0%), spleen–kidney deficiency (10/44, 22.7%), and yin deficiency (7/44, 15.9%) were the most frequently reported subtype descriptions, together accounting for 63.6% of all deficiency-subtype occurrences.

### 4.4 Interpretation of the literature-reported TCM pathogenesis

Taken together, the literature-reported syndrome distribution may be interpreted as a pattern of root deficiency with branch excess and coexistence of deficiency and excess. Under the broad-domain coding scheme, Deficiency was the most frequently represented broad category, whereas dampness, heat, stasis, and toxin constituted the major excess-related factors. The predominance of Dampness–Heat and Stasis–Toxin among composite patterns further suggests that these factors were commonly reported in combination rather than in isolation. Because the Deficiency domain aggregates multiple original subtype descriptions, its relative frequency may partly reflect the broader level of aggregation used in the primary coding scheme and should therefore be interpreted cautiously in comparison with more narrowly defined pathological elements such as dampness, heat, stasis, and toxin.

From a TCM perspective, the included literature commonly framed insufficiency of healthy qi as an underlying context, accompanied by dampness–heat, blood stasis, and toxin-related manifestations. These observations reflect literature-reported syndrome characteristics rather than patient-level epidemiological prevalence and provide a descriptive basis for the therapeutic principles summarized below.

### 4.5 Therapeutic principles and representative formulae/preparations

Consistent with the reported syndrome profile, therapeutic strategies described in the included literature were generally organized around the combined principles of healthy-qi reinforcement and pathogen elimination. Common approaches included reinforcing healthy qi, clearing heat and draining dampness, activating blood and resolving stasis, and clearing toxin or resolving pathogenic accumulation.

The included sources reported classical formulae, modified prescriptions, empirical or self-developed formulae, and modern TCM preparations applied according to syndrome characteristics and treatment context. Representative formulae and preparations and their evidence-relevance strata are summarized in Table 1. More detailed information on therapeutic principles, core botanical drugs or main materials, reported or predicted mechanisms, study/model context, evidence-relevance strata, and supporting references is provided in Supplementary Table S5. Taxonomic and source identification of the medicinal materials used in these representative formulae and preparations is provided in Supplementary Table S8. Their inclusion in this syndrome-oriented synthesis reflects their reported use in the source literature and should not, by itself, be interpreted as evidence of pharmacological efficacy or mechanistic validation. Pharmacological and mechanistic evidence is critically evaluated separately in Section 5.

**TABLE 1:** Representative TCM formulae and preparations reported in NMIBC-related or broader bladder cancer studies and their evidence-relevance strata.

Formula/preparation	Reported TCM or treatment context	Main reported findings	Evidence-relevance stratum(s)	Representative references
Zhibai Dihuang Decoction	Deficiency–heat; post-TURBT setting	Reported improvements in oxidative-stress status, immune indices, tumor markers, and quality of life; additional predictive analyses explored possible molecular mechanisms	A+ E	(Zhang et al., 2022; Suo et al., 2022; Zhao et al., 2023)
Si Jun Zi Decoction	Deficiency-dominant context	Induced apoptosis and enhanced macrophage-mediated antitumor activity in bladder cancer-bearing mice	C	(Li et al., 2002 ; 2005)
Buzhong Yiqi Decoction	Deficiency-dominant; postoperative intravesical-therapy setting	Reported improvements in immune indices and treatment response, with reductions in selected adverse reactions and VEGF levels	A	(Wang et al., 2021; Yan et al., 2024)
Bazheng San	Dampness–heat; postoperative adjunctive-treatment setting	Reported reductions in recurrence, treatment-related adverse reactions, and urinary symptoms, with improved quality of life	A	(Li, 2015; Luo et al., 2015; Pan and Zhang, 2021)
Longshe Yangquan Decoction	Dampness–heat–toxin/toxin-related context	Computational analyses predicted potential effects on proliferation, invasion, inflammation, and related biological processes	E	(Cheng et al., 2022)
Didang Decoction	Stasis/stasis–toxin context	Reported improvements in quality of life and NMIBC-related symptoms; *in vitro* inhibition of bladder cancer cell proliferation and additional predictive mechanistic findings were also reported	A+ C + E	(Shen, 2020; Shen et al., 2020)
Guizhi Fuling Wan	Stasis/stasis–toxin context	Inhibited bladder cancer growth and induced cell-cycle arrest/apoptosis in experimental models; an intravesical-relevant mouse model showed relatively low urothelial toxicity	B+ C	(Lu et al., 2013 ; Lu et al., 2016)
Fuzheng Xiaozheng Decoction	Deficiency with stasis–toxin; postoperative adjunctive-treatment setting	Reported improvements in selected immune indices and symptoms and reductions in tumor-related and invasion markers; experimental and predictive studies investigated proliferation-, migration-, and invasion-related effects	A+ C + E	(Zhuang et al., 2023; Yu et al., 2025; Yang et al., 2025)
Compound Kushen Injection	Toxin-related; investigated as an adjunct to intravesical or systemic treatment	Reported improvements in selected immune/inflammatory indices and treatment tolerance; experimental studies reported effects on proliferation, invasion, apoptosis, and autophagy	A+ C + D + E	(Zhang et al., 2017; Cao et al., 2017; Zhang et al., 2022; Zhang et al., 2024; Tian, 2025)
Huachansu	Toxin-related preparation	Induced apoptosis and inhibited migration/invasion in bladder cancer cell models	C	(Lü et al., 2010; Kang et al., 2017)

Evidence-relevance strata reflect study design and proximity to the NMIBC, clinical context rather than formal levels of evidence quality. Stratum A, direct NMIBC, clinical evidence; Stratum B, NMIBC-relevant translational evidence from postoperative, intravesical, orthotopic, reimplantation, or related models; Stratum C, experimental evidence from general bladder cancer models without specific NMIBC, relevance; Stratum D, broader bladder cancer clinical or meta-analytic evidence without separately identifiable NMIBC, data; and Stratum E, indirect or hypothesis-generating evidence derived from computational or predictive analyses, including network pharmacology, molecular docking, bioinformatics, and predictive transcriptomics. Multiple strata were assigned when supported by different studies. Reported TCM, syndrome or treatment contexts are presented descriptively and should not be interpreted as direct biological equivalents of specific molecular mechanisms.

## 5 Pharmacological and mechanistic evidence relevant to adjunctive TCM use in NMIBC

NMIBC recurrence and progression involve multiple interconnected biological processes, including dysregulated tumor-cell proliferation and survival, inflammatory and immune-microenvironmental remodeling, angiogenesis, invasion, migration, and treatment resistance. Key signaling networks implicated in NMIBC biology include PI3K/AKT/mTOR, FGFR3/RAS/MAPK, and VEGF-related pathways (Cheng et al., 2017; Schuettfort et al., 2021; Furuya et al., 2022; Glaviano et al., 2023; Grębowski et al., 2023; Tang et al., 2024; Yang et al., 2024; Wen et al., 2025).

Against this biological background, this section critically synthesizes pharmacological and mechanistic evidence concerning representative TCM formulae, preparations, plant-derived metabolites, and other defined natural products. The evidence is organized according to major biological processes relevant to bladder cancer and NMIBC, including tumor-cell proliferation and cell death, inflammatory and immune regulation, and angiogenesis, invasion, migration, and tumor-cell reimplantation. Because much of the available mechanistic evidence derives from broader bladder cancer models rather than NMIBC-specific studies, these findings are interpreted primarily as mechanistic clues unless supported by direct NMIBC clinical or experimental evidence.

### 5.1 Regulation of tumor-cell proliferation and cell death

Dysregulated tumor-cell proliferation and resistance to cell death are central features of bladder cancer biology. In the postoperative setting of NMIBC, survival of residual tumor cells may contribute to subsequent recurrence. Accordingly, apoptosis, ferroptosis, autophagy, oxidative stress, and cell-cycle regulation constitute major biological processes examined in studies of individual TCM formulae and preparations, plant-derived metabolites, and other defined natural products (Figure 3).

**FIGURE 3:**
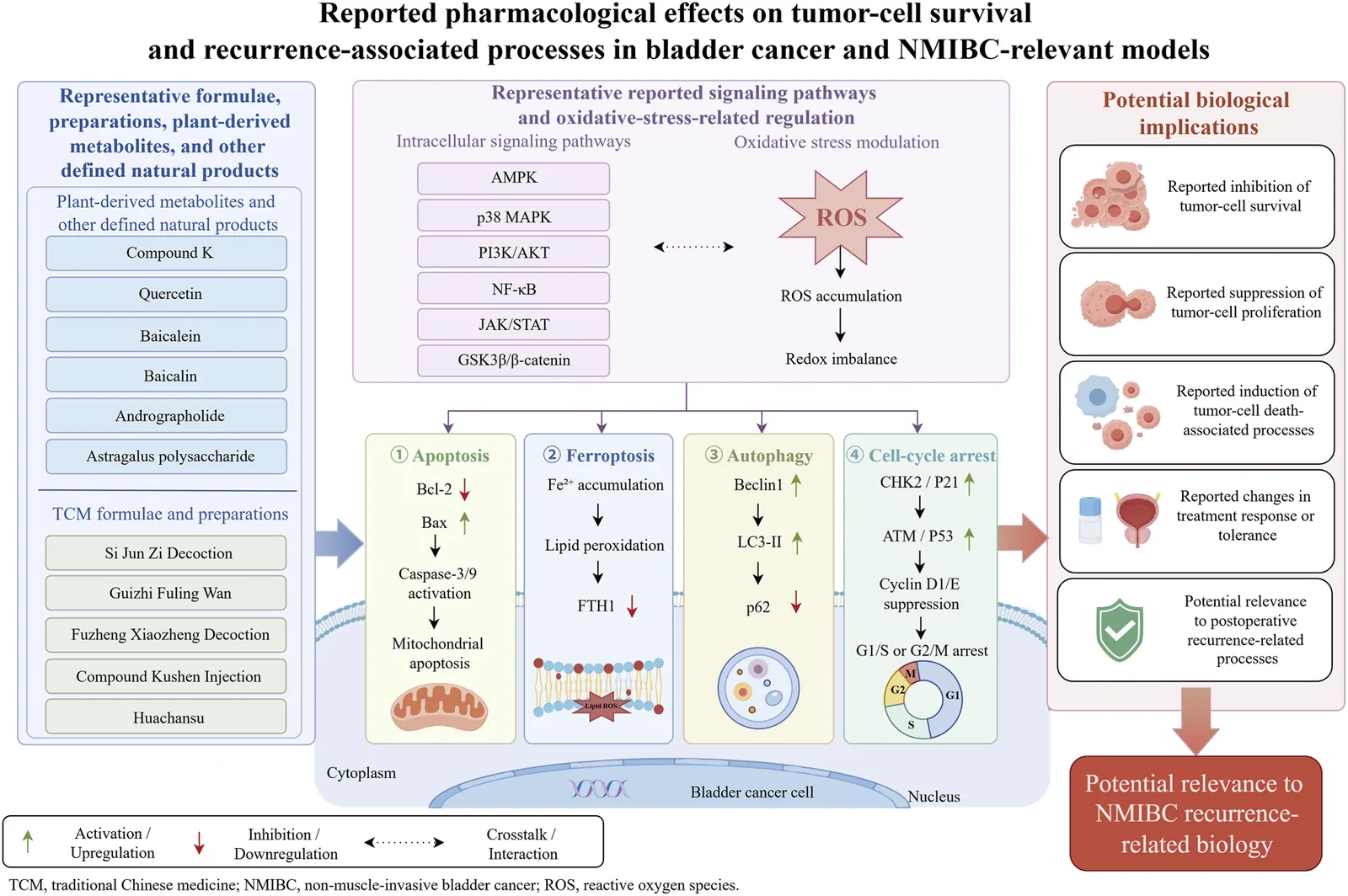
Reported pharmacological effects on tumor-cell survival and recurrence-associated processes in bladder cancer and NMIBC-relevant models. Figure legend: The figure summarizes findings from individual TCM formulae and preparations, plant-derived metabolites, and other defined natural products investigated in relation to apoptosis, ferroptosis, autophagy, oxidative stress, cell-cycle regulation, and related tumor-cell phenotypes. Evidence derives from heterogeneous experimental systems, including broader bladder cancer models, and should not be interpreted as demonstrating a unified pharmacological mechanism of TCM or a clinically established effect on NMIBC recurrence.

Several plant-derived metabolites and other defined natural products have been investigated for effects on cell-survival and cell-death pathways in bladder cancer models. Compound K, a major metabolite of ginsenosides, was reported to induce reactive oxygen species (ROS) accumulation and activate p38 MAPK signaling, thereby promoting mitochondria-dependent apoptosis in T24 bladder cancer cells (Wang et al., 2013). Quercetin inhibited proliferation and colony formation and induced apoptosis through AMPK-related regulation, 4E-BP1/S6K signaling, and reduced mutant p53 and Survivin expression (Ma et al., 2006; Su et al., 2016). Baicalein was reported to induce ROS-dependent caspase activation and mitochondrial apoptosis, whereas baicalin induced ferroptosis-related cell death through FTH1-associated mechanisms, ROS accumulation, and increased intracellular iron levels (Wu et al., 2013; Choi et al., 2016; Kong et al., 2021). Additional plant-derived metabolites and other natural products, including luteolin (Iida et al., 2020), glabridin (Yang et al., 2023), berberine (Xia et al., 2021), febrifugine (Chen et al., 2024), andrographolide (Deng et al., 2019; Xuan et al., 2022), astragalus polysaccharide, and paeonol (Zhang et al., 2021), have also been investigated in bladder cancer models and are summarized in Supplementary Table S6.

Evidence at the formula or preparation level is likewise predominantly preclinical. Si Jun Zi Decoction promoted apoptosis in bladder cancer-bearing mice in association with Fas/FasL upregulation and macrophage-related antitumor activity (Li et al., 2002; Li et al., 2005). Fuzheng Xiaozheng Decoction has been reported to inhibit proliferation and migration through Bcl-2/BAX- and GSK3β/β-catenin-related mechanisms (Zhuang et al., 2023; Yang et al., 2025), whereas Guizhi Fuling Wan induced cell-cycle arrest and apoptosis through CHK2/P21- and ATM/P53-related pathways in experimental studies (Lu et al., 2013; Lu et al., 2016). Compound Kushen Injection has also been investigated for effects on apoptosis, autophagy, and T24 cell proliferation (Zhang et al., 2020). Huachansu/Cinobufacin preparations have been investigated for pro-apoptotic effects in bladder cancer cell models (Lü et al., 2010), whereas Xiaozhiling has demonstrated growth-inhibitory and pro-apoptotic effects *in vitro*, although its specific mechanisms remain insufficiently characterized (Zhou et al., 2020).

Collectively, these studies provide preclinical evidence concerning the effects of individual formulae, preparations, plant-derived metabolites, and other defined natural products on tumor-cell survival and cell-death-related processes in bladder cancer models. However, these findings arise from distinct interventions and experimental systems and should not be interpreted as demonstrating a shared pharmacological effect of TCM as a therapeutic class. Moreover, they do not establish that the reported experimental effects translate into reduced postoperative recurrence in NMIBC. Further validation in NMIBC-relevant models and studies incorporating recurrence-related outcomes is therefore required.

### 5.2 Regulation of inflammatory responses and the immune microenvironment

Inflammatory and immune regulation is highly relevant to NMIBC because intravesical BCG depends on local immune activation and is strongly influenced by the tumor immune microenvironment (Ibrahim and Kalinski, 2024; Bardowska et al., 2025; Diamantidis et al., 2025; Wang et al., 2026). Regulatory T cells and other immunoregulatory components have been implicated in NMIBC progression and treatment response (Ibrahim and Kalinski, 2024; Bardowska et al., 2025; Diamantidis et al., 2025; Wang et al., 2026), while ERBB2 mutations have been associated with HIF-1 stabilization, tumor-cell proliferation, migration, invasion, and macrophage infiltration in recurrent NMIBC (Ibrahim and Kalinski, 2024; Bardowska et al., 2025; Diamantidis et al., 2025; Wang et al., 2026). These findings provide a biological context for evaluating inflammatory and immune-related outcomes (Figure 4).

**FIGURE 4:**
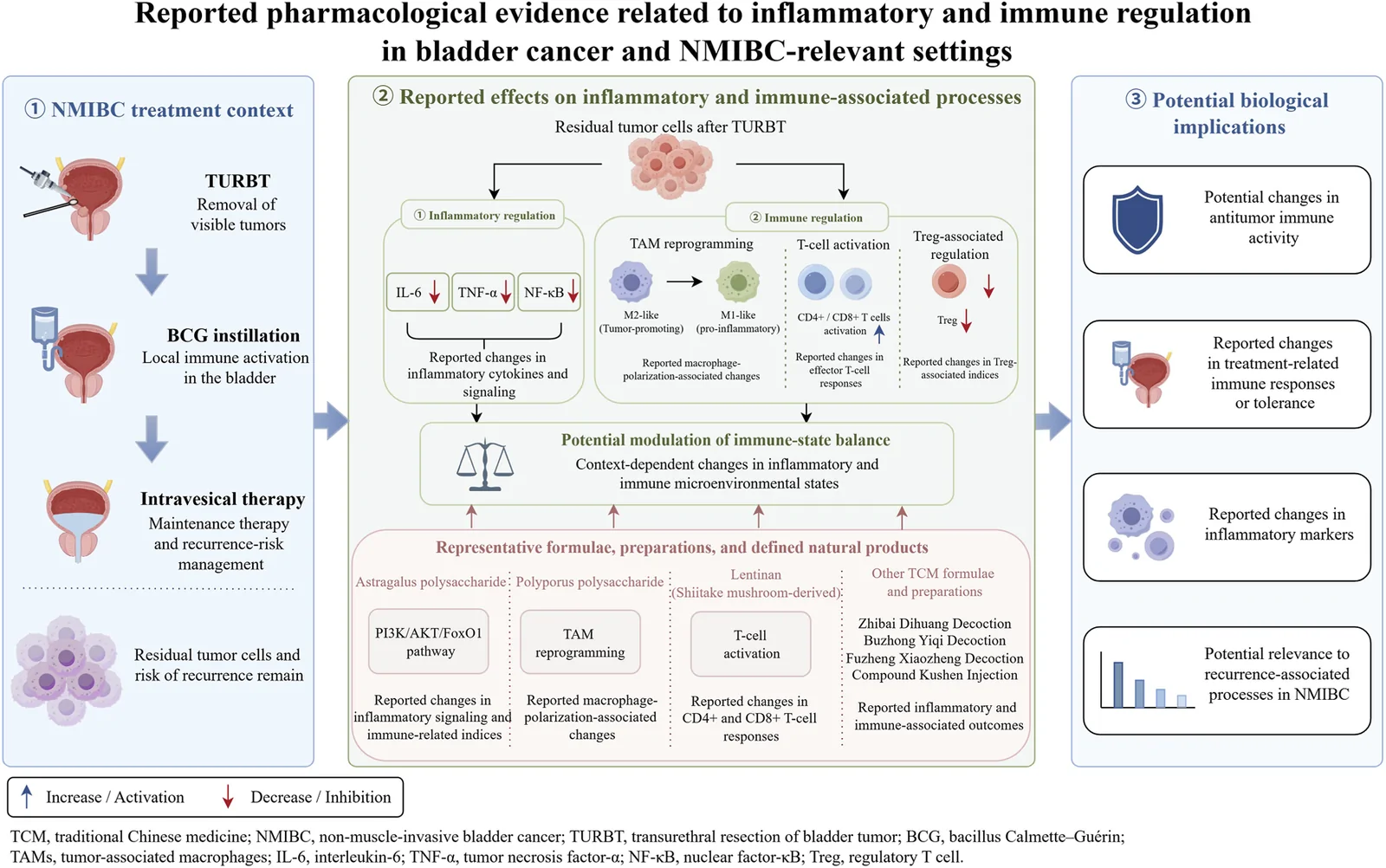
Reported pharmacological evidence related to inflammatory and immune regulation in bladder cancer and NMIBC-relevant settings. Figure legend: The figure summarizes findings from individual TCM formulae and preparations and defined natural products investigated in relation to inflammatory signaling, macrophage polarization, T-cell responses, cytokine regulation, and treatment-related immune outcomes. Evidence derives from heterogeneous clinical and experimental settings, including broader bladder cancer and tumor-immunology models. Immune-associated changes should not be interpreted as uniformly beneficial, because immune activation may coexist with compensatory immunosuppression or checkpoint-mediated adaptive resistance. Accordingly, the figure does not establish a unified immunomodulatory mechanism of TCM or a clinically validated effect on BCG response or NMIBC recurrence.

Several defined natural products, particularly polysaccharides, have been investigated for immunomodulatory effects in bladder cancer models. Astragalus polysaccharide has been reported to inhibit bladder cancer cell proliferation and enhance chemosensitivity through PI3K/AKT/FoxO1-related signaling (Tong et al., 2023; Chen et al., 2025). Polyporus polysaccharides and related fractions have been associated with changes in the tumor immune microenvironment, including macrophage polarization, with possible involvement of NF-κB/NLRP3-related signaling (Zhang et al., 2015; Jia et al., 2021; Liu et al., 2022). Lentinan has also been reported to modulate T-cell responses, immunosuppressive-cell populations, and cytokine profiles in experimental settings (Sun et al., 2020). These findings remain primarily preclinical.

At the formula and preparation level, modified Buzhong Yiqi Decoction combined with intravesical therapy has been associated with changes in selected immune-related outcomes and treatment-related adverse reactions in patients with NMIBC (Suo et al., 2022; Zhao et al., 2023; Yan et al., 2024). Modified Zhibai Dihuang Decoction combined with pirarubicin has also been associated with changes in immune function, tumor-related markers, and quality of life (Suo et al., 2022), whereas Fuzheng Xiaozheng Decoction has been evaluated in postoperative NMIBC and in experimental or predictive studies involving inflammation- and invasion-related networks (Yu et al., 2025). Compound Kushen Injection has likewise been investigated in clinical and experimental settings, with reported changes in selected inflammatory factors, immune indices, treatment tolerance, and macrophage-associated signaling (Cao et al., 2017; Zhang et al., 2024; Tian, 2025).

Overall, evidence from individual formulae, preparations, and defined natural products suggests possible modulation of inflammatory or immune-related outcomes, but direct evidence regarding recurrence, progression, BCG response, or intratumoral immune remodeling remains limited. Importantly, immune activation should not automatically be interpreted as improved antitumor immunity. In NMIBC, activated immune states may coexist with compensatory immunosuppression and checkpoint-mediated adaptive resistance, including PD-1/PD-L1-associated patterns in BCG-treated disease. Therefore, changes in cytokines, lymphocyte subsets, macrophage polarization, or related immune indices may be context dependent and cannot be assumed to enhance BCG efficacy; their functional consequences may be neutral or potentially unfavorable. Direct functional and clinical validation is required before such immune changes can be interpreted as therapeutically beneficial.

### 5.3 Regulation of angiogenesis, invasion, migration, and tumor-cell reimplantation

Angiogenesis, extracellular-matrix remodeling, invasion, and migration are important processes in bladder cancer progression and may contribute to local recurrence. In NMIBC, tumor-cell adhesion and reimplantation after TURBT represent an additional recurrence-related process. Evidence concerning angiogenesis, epithelial–mesenchymal transition (EMT), matrix degradation, migration, and reimplantation is therefore particularly relevant (Figure 5).

**FIGURE 5:**
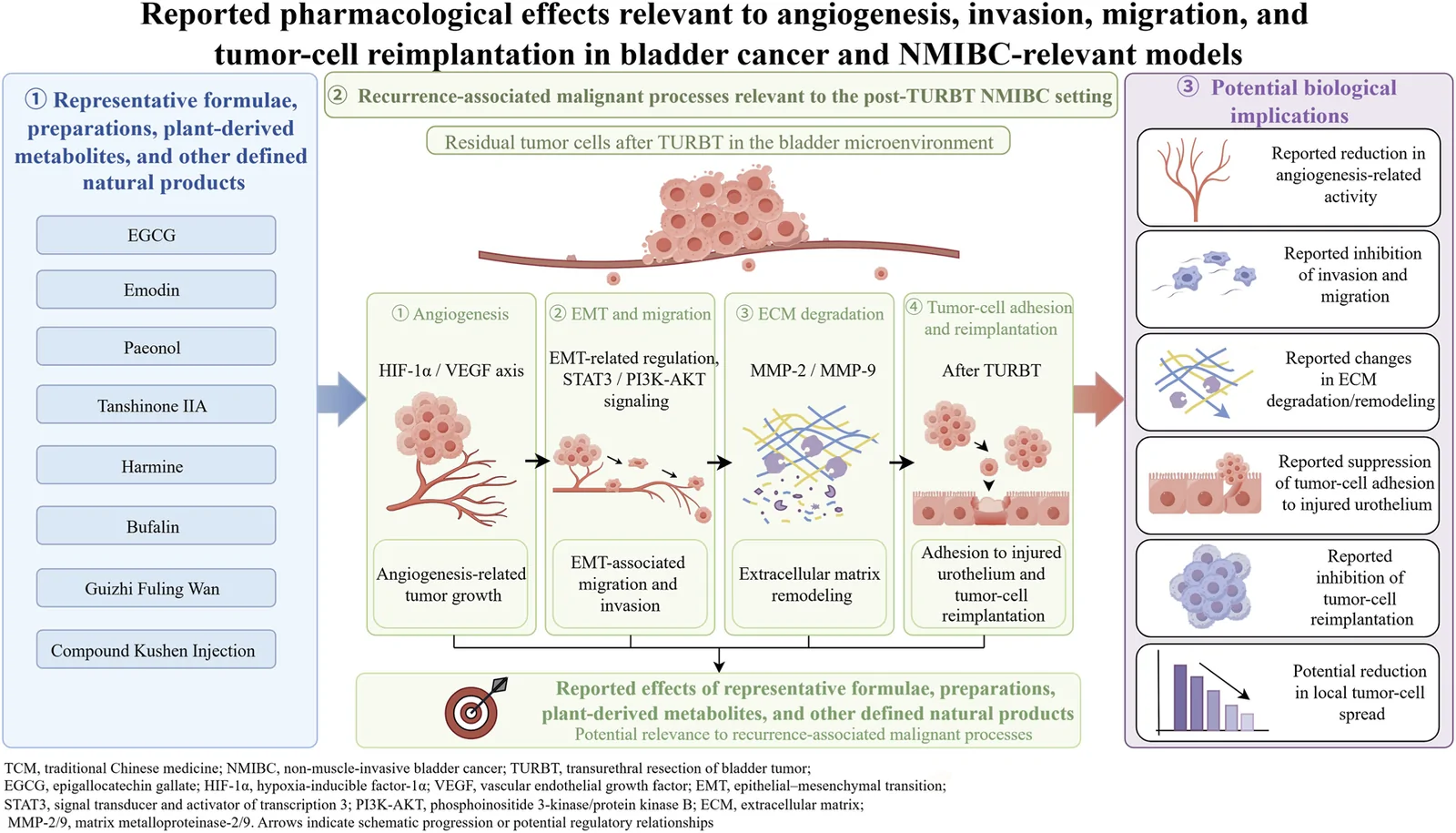
Reported pharmacological effects relevant to angiogenesis, invasion, migration, and tumor-cell reimplantation in bladder cancer and NMIBC-relevant models. Figure legend: The figure summarizes findings from individual TCM formulae and preparations, plant-derived metabolites, and other defined natural products investigated in relation to angiogenesis, epithelial–mesenchymal transition-associated migration, extracellular-matrix remodeling, invasion, and tumor-cell adhesion or reimplantation. Evidence derives from heterogeneous experimental systems, including broader bladder cancer models, and should be interpreted as mechanistic or translational evidence rather than as demonstrating a unified pharmacological effect of TCM or a clinically established reduction in NMIBC recurrence.

Several plant-derived metabolites and other defined natural products have been investigated for anti-angiogenic, anti-invasive, or anti-migratory effects in bladder cancer models. Emodin was associated with reduced invasion and angiogenesis-related signaling involving Notch1, VEGF, and MMP-2 (Ma et al., 2017). Bufalin was reported to inhibit the migration and invasion of T24 bladder carcinoma cells, with effects involving matrix metalloproteinases and tight-junction regulation (Hong et al., 2013). Tanshinone IIA showed anti-migratory and anti-invasive effects involving STAT3-related signaling (Huang et al., 2017; Hai-rong et al., 2019), while harmine demonstrated anti-angiogenic activity involving VEGF/VEGFR2 signaling in experimental models (Huang et al., 2017; Hai-rong et al., 2019). These findings remain predominantly preclinical and derive largely from general bladder cancer models.

EGCG is of particular relevance to tumor-cell reimplantation. In a model of injured bladder mucosa, intravesical EGCG reduced adhesion and implantation of transitional cell carcinoma cells, with an effect comparable to mitomycin C within that experimental setting (Kemberling et al., 2003). Subsequent studies reported effects on migration, extracellular-matrix degradation, and invasion involving MMP-9 and VEGF regulation (Lee et al., 2019; Piwowarczyk et al., 2020). Although these findings do not establish clinical efficacy, the intravesical reimplantation model more closely reflects a postoperative NMIBC recurrence-related process than conventional proliferation assays.

At the formula and preparation level, Longshe Yangquan Decoction, Zhibai Dihuang Decoction, and Fuzheng Xiaozheng Decoction have been investigated using experimental or predictive approaches involving proliferation, migration, inflammation, hypoxia, and angiogenesis-related pathways, including VEGF, HIF-1α, and NF-κB (Cheng et al., 2022; Zhang et al., 2022; Yu et al., 2025). Findings derived from network pharmacology or other predictive analyses should be regarded as hypothesis-generating unless experimentally validated. Compound Kushen Injection and Magui Liquid have also been investigated for effects on migration and related signaling in bladder cancer models (Zhang et al., 2017; Zhang et al., 2022; Mao et al., 2025; Zhao et al., 2026).

Guizhi Fuling Wan inhibited tumor growth in an orthotopic bladder cancer mouse model and showed a tumor-suppressive trend comparable to BCG and mitomycin C within that experimental setting, with relatively low local irritation and tissue toxicity (Lu et al., 2016). This finding supports further investigation of local bladder-directed applications but should not be interpreted as evidence of comparable clinical efficacy.

Overall, individual formulae, preparations, plant-derived metabolites, and other defined natural products have been investigated for effects on angiogenesis, extracellular-matrix remodeling, invasion, migration, and tumor-cell reimplantation. However, these findings arise from heterogeneous experimental systems and do not establish a shared pharmacological effect of TCM as a therapeutic class or a clinically validated effect on NMIBC recurrence.

### 5.4 Integrative interpretation and limitations of the mechanistic evidence

Taken together, individual formulae, preparations, plant-derived metabolites, and other defined natural products have been investigated in separate studies for effects on biological processes relevant to bladder cancer, including tumor-cell survival and death, inflammatory and immune regulation, angiogenesis, extracellular-matrix remodeling, invasion, migration, and reimplantation. These observations arise from heterogeneous interventions, experimental systems, and levels of evidence and do not establish a unified pharmacological mechanism for TCM as a therapeutic class or an NMIBC-specific multi-target mechanism. The evidence supporting individual targets and pathways also varies substantially in methodological strength.

The distinction between experimentally demonstrated and hypothesis-generating mechanisms is particularly important. Network pharmacology, molecular docking, bioinformatic prediction, and related approaches may be useful for identifying candidate targets and pathways, but such findings require independent experimental validation before they can be considered established mechanisms. Conventional 2D bladder cancer cell-line models can demonstrate effects on individual cellular or molecular processes but cannot reproduce key features of the postoperative NMIBC setting, including residual disease after TURBT, tumor-cell reimplantation, intravesical treatment exposure, and the local immune microenvironment; such findings therefore cannot directly establish effects on recurrence, progression, or BCG response. Findings from animal models likewise require cautious interpretation according to their disease context and the extent to which they reproduce clinically relevant NMIBC conditions.

Pharmacokinetic limitations further constrain translation of isolated-metabolite findings. Human pharmacokinetic studies indicate that systemic exposure to several plant-derived metabolites discussed in this review is strongly influenced by chemical form, food matrix, and metabolism. Quercetin bioavailability varies according to glycoside structure and plant matrix, with circulating quercetin detected predominantly as conjugated metabolites (Graefe et al., 2001). EGCG exposure is likewise affected by dosing conditions; in healthy volunteers, fasting substantially increased plasma EGCG exposure compared with administration with food (Chow et al., 2005). After oral administration of artichoke leaf extracts, luteolin glycosides were recovered in plasma and urine as sulfate or glucuronide conjugates rather than as free luteolin or intact glycosides (Wittemer et al., 2005). Consequently, concentrations producing biological effects in cell-based experiments may not correspond to those achievable after oral administration of multi-botanical-drug formulae, and circulating metabolites may differ substantially from the parent molecules tested *in vitro*. Local intravesical administration, as investigated for EGCG in bladder models, represents a distinct exposure context and should not be interpreted as pharmacokinetically equivalent to oral administration. These differences should be considered when extrapolating experimental findings to clinical TCM use.

Clinical evidence also remains limited. Existing studies have predominantly assessed symptoms, peripheral immune or inflammatory indices, treatment-related adverse effects, short-term treatment responses, or quality of life, whereas recurrence-free survival, progression-free survival, time to recurrence, and bladder preservation have rarely been evaluated as primary endpoints. Experimental studies have also incompletely modeled NMIBC-specific clinical scenarios such as residual disease after TURBT, tumor-cell reimplantation, and exposure to intravesical therapies.

Future mechanistic studies should therefore prioritize standardized characterization and quality control of TCM formulae and preparations, experimental validation of proposed target–pathway relationships, use of NMIBC-relevant experimental models, pharmacokinetic characterization of clinically relevant exposures, and integration of clinically meaningful recurrence-related endpoints. Such approaches will be necessary to determine which pharmacological observations can be translated from broader bladder cancer models into credible evidence for adjunctive NMIBC management.

## 6 Clinical relevance, evidence gaps, and future directions

### 6.1 Potential clinical value of adjunctive TCM in NMIBC management

The potential clinical relevance of TCM in NMIBC lies primarily in its adjunctive use within standard care. Given the need for repeated intravesical treatment and long-term surveillance, TCM may have a supportive role in symptom management, treatment tolerance, and quality of life. However, available clinical findings on immune or inflammatory indices and quality-of-life outcomes should not be interpreted as evidence of reduced recurrence or progression without direct recurrence-related endpoints.

Adjunctive TCM should therefore be evaluated within specific treatment regimens rather than as an alternative to established or emerging NMIBC therapies. Particular attention should be paid to treatment tolerance, pharmacokinetic and pharmacodynamic interactions, and treatment-related immune effects. Current evidence does not establish a unified NMIBC-specific pharmacological mechanism or demonstrate improved recurrence or progression outcomes.

The combination of TCM-related interventions with emerging systemic therapies also warrants investigation. Preclinical bladder cancer studies have reported antitumor effects when bisdemethoxycurcumin or astragaloside IV was combined with anti-PD-L1 therapy (Shao et al., 2017; Kong et al., 2024), but these findings remain preclinical and do not establish benefit in NMIBC patients. Direct evidence for TCM combined with antibody–drug conjugates (ADCs) is currently lacking. Future studies should assess such combinations with attention to efficacy, pharmacological interactions, overlapping toxicities, and clinically meaningful oncological outcomes.

### 6.2 Major limitations and evidence gaps

Several limitations constrain interpretation and clinical translation of the current evidence.

First, syndrome-related research remains methodologically heterogeneous, with inconsistent terminology, diagnostic criteria, classification methods, and outcome assessment, limiting comparability and reproducibility across studies. Clinical heterogeneity further constrains interpretation, as the included sources varied in reported disease stage, grade or risk category, treatment phase, and intravesical treatment exposure, while these characteristics were incompletely reported in many studies. Consequently, the observed syndrome distributions should not be assumed to apply uniformly across different NMIBC clinical subgroups or treatment settings.

Second, clinical evidence is generally limited by small or single-center studies, incomplete methodological reporting, and reliance on symptoms, immune or inflammatory indices, short-term responses, and quality-of-life measures rather than recurrence-free survival, progression-free survival, or other long-term oncological endpoints.

Third, a substantial translational gap remains between clinical TCM use and mechanistic research. TCM is typically administered as multi-botanical-drug formulae or preparations, whereas mechanistic studies frequently examine isolated metabolites or other defined natural products or rely on hypothesis-generating approaches. Direct links among clinically used interventions, experimentally validated mechanisms, and meaningful NMIBC outcomes therefore remain limited.

Fourth, intervention characterization and quality control remain inconsistent. Reporting of composition and dose, preparation and administration, material authentication, product traceability, and phytochemical characterization varied substantially across studies (Supplementary Table S7), limiting reproducibility.

Fifth, safety requires explicit consideration. Aristolochic acids are recognized human carcinogens, and botanical products containing *Aristolochia* species have been classified as carcinogenic to humans (Iarc Working Group On The Evaluation Of Carcinogenic Risks To Humans, 2002; National Toxicology Program, 2021). Although the interventions reviewed here should not be assumed to contain aristolochic acids without documented exposure, rigorous botanical authentication, traceable sourcing, and exclusion of potential aristolochic-acid sources remain essential.

Finally, most mechanistic evidence derives from conventional bladder cancer cell or animal models rather than NMIBC-specific settings. Post-TURBT residual disease, intravesical exposure, tumor-cell reimplantation, and recurrence after local treatment remain insufficiently modeled, and extrapolation to NMIBC should therefore be cautious.

### 6.3 Future research directions

Future research should prioritize syndrome standardization, reproducible intervention characterization, mechanistic validation, and clinically meaningful evaluation.

For syndrome-related research, standardized terminology and transparent criteria for syndrome-element deconstruction and classification are needed to improve reproducibility and cross-study comparability. Prospective studies should further examine whether syndrome characteristics vary by disease risk, pathological features, treatment stage, or longitudinal course.

Mechanistic studies should avoid assuming fixed correspondences between TCM syndromes and molecular pathways. Clearly characterized formulae, preparations, plant-derived metabolites, and other defined natural products should be evaluated using experimentally validated approaches, while network pharmacology, molecular docking, bioinformatics, and omics analyses should be treated as hypothesis-generating until validated in relevant experimental or patient-derived models.

Advanced systems such as organoids, patient-derived samples, and spatial or single-cell omics may help determine whether findings from conventional models are reproduced in biologically relevant NMIBC settings. Associations between TCM syndrome characteristics and molecular, metabolic, or microenvironmental profiles may also be explored, but should be interpreted as hypotheses rather than established biological equivalences.

Molecular heterogeneity should be incorporated into future evaluation of adjunctive TCM. UROMOL2021 classifies NMIBC into molecular classes 1, 2a, 2b, and 3 with distinct genomic, transcriptomic, immune, and prognostic features (Lindskrog et al., 2021). No study identified in this review evaluated differential responses to TCM across these classes. Future studies should therefore assess pharmacological and clinical responses within molecularly defined NMIBC subgroups without equating molecular subtypes with TCM syndrome categories.

Clinically, well-designed prospective and multicenter studies should evaluate adjunctive TCM within standard NMIBC treatment, with greater emphasis on recurrence-free survival, progression-free survival, time to first recurrence, bladder preservation, and treatment tolerance. Stratification by molecular subtype, NMIBC risk category, treatment modality, and treatment stage may help identify settings in which adjunctive TCM provides measurable benefit.

## 7 Conclusion

The scoping review identified deficiency, dampness, heat, stasis, and toxin as the most frequently reported syndrome elements in NMIBC-related literature, with Dampness–Heat and Stasis–Toxin among the most frequently reported composite patterns. These findings describe literature-level reporting characteristics rather than patient-level prevalence and provide a structured summary of the TCM syndrome profile reported in NMIBC.

Separately, the pharmacological and mechanistic synthesis identified clinical, experimental, and predictive evidence concerning selected TCM formulae, preparations, plant-derived metabolites, and other defined natural products investigated in relation to tumor-cell survival and death, inflammatory and immune regulation, angiogenesis, invasion, migration, and tumor-cell reimplantation. However, much of this evidence derives from broader bladder cancer models or hypothesis-generating analyses and does not establish NMIBC-specific mechanisms or reduced recurrence and progression. Future studies should prioritize standardized syndrome reporting, reproducible characterization and quality control of TCM interventions, NMIBC-relevant experimental models, and clinically meaningful recurrence- and progression-related outcomes.
