# Supplementary material for: Traditional Chinese medicine in non-muscle-invasive bladder cancer: a scoping review of syndrome characteristics and critical synthesis of pharmacological and mechanistic evidence
Source: Front Pharmacol. 2026 Sep 22;17:1921622. doi: 10.3389/fphar.2026.1921622 (PMC13639432; doi:10.3389/fphar.2026.1921622)
Supplement: Supplementary file 3 [file Table1.docx]

Table 1. Representative TCM formulae and preparations reported in NMIBC-related or broader bladder cancer studies and their evidence-relevance strata.

| **Formula/preparation** | **Reported TCM or treatment context** | **Main reported findings** | **Evidence-relevance stratum(s)** | **Representative references** |
| --- | --- | --- | --- | --- |
| Zhibai Dihuang Decoction | Deficiency–heat; post-TURBT setting | Reported improvements in oxidative-stress status, immune indices, tumor markers, and quality of life; additional predictive analyses explored possible molecular mechanisms | A + E | (Zhang et al., 2022; Suo et al., 2022; Zhao et al., 2023) |
| Si Jun Zi Decoction | Deficiency-dominant context | Induced apoptosis and enhanced macrophage-mediated antitumor activity in bladder cancer-bearing mice | C | ( Li et al., 2002, 2005) |
| Buzhong Yiqi Decoction | Deficiency-dominant; postoperative intravesical-therapy setting | Reported improvements in immune indices and treatment response, with reductions in selected adverse reactions and VEGF levels | A | (Wang et al., 2021; Yan et al., 2024) |
| Bazheng San | Dampness–heat; postoperative adjunctive-treatment setting | Reported reductions in recurrence, treatment-related adverse reactions, and urinary symptoms, with improved quality of life | A | ( Li, 2015; Luo et al., 2015; Pan and Zhang, 2021) |
| Longshe Yangquan Decoction | Dampness–heat–toxin/toxin-related context | Computational analyses predicted potential effects on proliferation, invasion, inflammation, and related biological processes | E | (Cheng et al., 2022) |
| Didang Decoction | Stasis/stasis–toxin context | Reported improvements in quality of life and NMIBC-related symptoms; in vitro inhibition of bladder cancer cell proliferation and additional predictive mechanistic findings were also reported | A + C + E | (Shen, 2020; Shen et al., 2020) |
| Guizhi Fuling Wan | Stasis/stasis–toxin context | Inhibited bladder cancer growth and induced cell-cycle arrest/apoptosis in experimental models; an intravesical-relevant mouse model showed relatively low urothelial toxicity | B + C | (Lu et al., 2013, 2016) |
| Fuzheng Xiaozheng Decoction | Deficiency with stasis–toxin; postoperative adjunctive-treatment setting | Reported improvements in selected immune indices and symptoms and reductions in tumor-related and invasion markers; experimental and predictive studies investigated proliferation-, migration-, and invasion-related effects | A + C + E | (Zhuang et al., 2023; Yu et al., 2025; Yang et al., 2025) |
| Compound Kushen Injection | Toxin-related; investigated as an adjunct to intravesical or systemic treatment | Reported improvements in selected immune/inflammatory indices and treatment tolerance; experimental studies reported effects on proliferation, invasion, apoptosis, and autophagy | A + C + D + E | ( Zhang et al., 2017; Cao et al., 2017; Zhang et al., 2022; Zhang et al., 2024; Tian, 2025) |
| Huachansu | Toxin-related preparation | Induced apoptosis and inhibited migration/invasion in bladder cancer cell models | C | ( Lüet al., 2010; Kang et al., 2017) |

**Note:** Evidence-relevance strata reflect study design and proximity to the NMIBC clinical context rather than formal levels of evidence quality. Stratum A, direct NMIBC clinical evidence; Stratum B, NMIBC-relevant translational evidence from postoperative, intravesical, orthotopic, reimplantation, or related models; Stratum C, experimental evidence from general bladder cancer models without specific NMIBC relevance; Stratum D, broader bladder cancer clinical or meta-analytic evidence without separately identifiable NMIBC data; and Stratum E, indirect or hypothesis-generating evidence derived from computational or predictive analyses, including network pharmacology, molecular docking, bioinformatics, and predictive transcriptomics. Multiple strata were assigned when supported by different studies. Reported TCM syndrome or treatment contexts are presented descriptively and should not be interpreted as direct biological equivalents of specific molecular mechanisms.
