# Supplementary material for: Traditional Chinese medicine in non-muscle-invasive bladder cancer: a scoping review of syndrome characteristics and critical synthesis of pharmacological and mechanistic evidence
Source: Front Pharmacol. 2026 Sep 22;17:1921622. doi: 10.3389/fphar.2026.1921622 (PMC13639432; doi:10.3389/fphar.2026.1921622)
Supplement: Supplementary file 4 [file Table3.docx]

Supplementary Table S 2. Full electronic search strategies for the scoping-review component

| Final searches were conducted on 5 August 2026. Publication date was restricted to 1 January 2000–31 December 2025. No language or publication-type restrictions were applied at the search stage. | | | | | |
| --- | --- | --- | --- | --- | --- |
| Database / platform | Search field | Full search strategy | Limits | Date searched | Records retrieved |
| CNKI | Subject | ("膀胱癌" OR "人膀胱癌" OR "表浅性膀胱癌" OR "浅表膀胱癌" OR "表浅膀胱癌" OR "非肌层浸润性膀胱癌" OR "非肌层浸润膀胱癌" OR "尿路上皮癌" OR "膀胱尿路上皮癌" OR "膀胱肿瘤" OR "膀胱肿瘤切除术" OR "膀胱肿瘤电切术") AND ("中医" OR "中医药" OR "中医治疗" OR "中医药治疗" OR "中医药疗法") | Publication date: 2000-01-01 to 2025-12-31; no language restriction; no publication-type restriction | 2026-08-05 | 213 |
| Wanfang Data | Subject | ("非肌层浸润性膀胱癌" OR "非肌层浸润膀胱癌" OR "浅表性膀胱癌" OR "浅表膀胱癌" OR "表浅性膀胱癌" OR "膀胱癌" OR "膀胱肿瘤") AND ("中医" OR "中医药" OR "中医治疗" OR "中医药治疗" OR "中医药疗法" OR "中药" OR "中药治疗") | Publication date: 2000-01-01 to 2025-12-31; no language restriction; no publication-type restriction | 2026-08-05 | 716 |
| PubMed | MeSH + Title/Abstract | (("Urinary Bladder Neoplasms"[Mesh] OR bladder cancer[Title/Abstract] OR bladder carcinoma[Title/Abstract] OR urothelial carcinoma[Title/Abstract] OR bladder tumor[Title/Abstract] OR bladder tumour[Title/Abstract] OR non-muscle invasive bladder cancer[Title/Abstract] OR NMIBC[Title/Abstract] OR superficial bladder cancer[Title/Abstract]) AND ("Medicine, Chinese Traditional"[Mesh] OR traditional Chinese medicine[Title/Abstract] OR Chinese medicine[Title/Abstract] OR Chinese herbal medicine[Title/Abstract] OR Chinese herb*[Title/Abstract] OR Chinese herbal formula*[Title/Abstract] OR Chinese patent medicine[Title/Abstract] OR TCM[Title/Abstract])) | Publication date: 2000-01-01 to 2025-12-31; no language restriction; no publication-type restriction | 2026-08-05 | 172 |
| Web of Science Core Collection | Topic (TS) | TS=("bladder cancer" OR "bladder carcinoma" OR "urothelial carcinoma" OR "bladder urothelial carcinoma" OR "bladder tumor" OR "bladder tumour" OR "bladder neoplasm*" OR "urinary bladder neoplasm*" OR "non-muscle invasive bladder cancer" OR "non-muscle-invasive bladder cancer" OR NMIBC OR "superficial bladder cancer") AND TS=("traditional Chinese medicine" OR "Chinese medicine" OR "Chinese herbal medicine" OR "Chinese herb*" OR "Chinese herbal formula*" OR "Chinese patent medicine" OR TCM) | Publication date: 2000-01-01 to 2025-12-31; no language restriction; no publication-type restriction | 2026-08-05 | 265 |
| Total records identified |  |  |  |  | 1366 |

Abbreviations: CNKI, China National Knowledge Infrastructure; NMIBC, non-muscle-invasive bladder cancer; TCM, traditional Chinese medicine; TS, topic.
