# Supplementary material for: Traditional Chinese medicine in non-muscle-invasive bladder cancer: a scoping review of syndrome characteristics and critical synthesis of pharmacological and mechanistic evidence
Source: Front Pharmacol. 2026 Sep 22;17:1921622. doi: 10.3389/fphar.2026.1921622 (PMC13639432; doi:10.3389/fphar.2026.1921622)
Supplement: Supplementary file 5 [file Table4.docx]

Supplementary Table S 3. Stratified syndrome-element reporting patterns by source type

| Source type was classified according to the Evidence basis field in Supplementary Table S1. | | | | | | |
| --- | --- | --- | --- | --- | --- | --- |
| **Syndrome element** | **All sources**  **(N = 59)** | | **Patient-based empirical**  **(N = 51)** | | **Experience-based**  **(N = 8)** | |
|  | **n** | **%** | **n** | **%** | **n** | **%** |
| Deficiency | 37 | 23.9% | 31 | 22.8% | 6 | 31.6% |
| Dampness | 33 | 21.3% | 28 | 20.6% | 5 | 26.3% |
| Heat | 32 | 20.6% | 29 | 21.3% | 3 | 15.8% |
| Stasis | 26 | 16.8% | 24 | 17.6% | 2 | 10.5% |
| Toxin | 23 | 14.8% | 22 | 16.2% | 1 | 5.3% |
| Qi stagnation | 3 | 1.9% | 1 | 0.7% | 2 | 10.5% |
| Phlegm | 1 | 0.6% | 1 | 0.7% | 0 | 0.0% |
| **Total syndrome-element occurrences** | **155** | **100.0%** | **136** | **100.0%** | **19** | **100.0%** |

Note: n denotes source-level syndrome-element occurrences. Each source contributed at most once to a given syndrome-element category. Percentages were calculated within each source-type stratum as the number of occurrences of a given syndrome element divided by the total number of syndrome-element occurrences in that stratum × 100%. Because individual sources could report multiple syndrome elements, occurrence totals exceed the number of sources. This stratified analysis was descriptive; no inferential between-group statistical testing was performed.
