# Supplementary material for: Traditional Chinese medicine in non-muscle-invasive bladder cancer: a scoping review of syndrome characteristics and critical synthesis of pharmacological and mechanistic evidence
Source: Front Pharmacol. 2026 Sep 22;17:1921622. doi: 10.3389/fphar.2026.1921622 (PMC13639432; doi:10.3389/fphar.2026.1921622)
Supplement: Supplementary file 6 [file Table5.docx]

Supplementary Table S 4. Sensitivity analysis of the Deficiency domain: distribution of original deficiency-related subtype descriptions

| **Original deficiency-related subtype/description** | **n** | **Frequency (%)** |
| --- | --- | --- |
| Qi deficiency | 11 | 25.0 |
| Spleen–kidney deficiency | 10 | 22.7 |
| Yin deficiency | 7 | 15.9 |
| Healthy-qi deficiency | 2 | 4.5 |
| Kidney deficiency | 2 | 4.5 |
| Kidney-yin deficiency | 2 | 4.5 |
| Spleen deficiency | 2 | 4.5 |
| Spleen-qi deficiency | 2 | 4.5 |
| Liver–kidney yin deficiency | 1 | 2.3 |
| Qi–blood deficiency | 1 | 2.3 |
| Qi–yin deficiency | 1 | 2.3 |
| Spleen–kidney qi deficiency | 1 | 2.3 |
| Spleen–kidney yang deficiency | 1 | 2.3 |
| Spleen–stomach deficiency | 1 | 2.3 |
| **Total deficiency-subtype occurrences** | **44** | **100.0** |

Note: In the primary syndrome-element analysis, all deficiency-related descriptions were grouped under the broad **Deficiency** domain to maintain a consistent hierarchical level across syndrome elements. Among the 37 sources contributing to the Deficiency domain, 44 original deficiency-subtype occurrences were identified because individual sources could report more than one deficiency subtype. Deficiency-subtype frequency was calculated as the number of occurrences of a given subtype divided by the total number of deficiency-subtype occurrences (n = 44) × 100%. Original subtype descriptions were retained without further collapsing. Percentages may not sum exactly to 100% because of rounding.
