# Supplementary material for: Traditional Chinese medicine in non-muscle-invasive bladder cancer: a scoping review of syndrome characteristics and critical synthesis of pharmacological and mechanistic evidence
Source: Front Pharmacol. 2026 Sep 22;17:1921622. doi: 10.3389/fphar.2026.1921622 (PMC13639432; doi:10.3389/fphar.2026.1921622)
Supplement: Supplementary file 7 [file Table6.docx]

Supplementary Table S 5. Detailed evidence summary of representative TCM formulae and preparations, including quantitative findings, study/model context, and evidence-relevance strata.

| **Therapeutic principle** | **Formula/preparation** | **Reported TCM/treatment context** | **Core botanical drugs or main materials** | **Reported findings** | **Representative quantitative findings** | **Reported/predicted mechanisms or pathways** | **Study/model context** | **Evidence-relevance stratum(s)** | **References** |
| --- | --- | --- | --- | --- | --- | --- | --- | --- | --- |
| Healthy-qi reinforcement | Zhibai Dihuang Decoction | Deficiency–heat; post-TURBT setting | Rehmanniae Radix Praeparata, Corni Fructus, Dioscoreae Rhizoma, Alismatis Rhizoma, Moutan Cortex, Poria, Anemarrhenae Rhizoma, Phellodendri Chinensis Cortex | Reported improvements in oxidative-stress status, immune indices, tumor markers, and quality of life | **n=92:** KPS↑; CD3+, CD4+, CD4+/CD8+, NK↑; CD44, CK20, BTA↓; MDA↓, SOD/AOA↑ (wk 6–9, all P<0.05; exact values NR).  **n=102:** response 90.2% vs 72.5%; adverse events 23.5% vs 54.9%; symptom scores, CA125/CEA↓; CD3+, CD4+, CD4+/CD8+ and QoL↑ (all P<0.05). | Oxidative-stress and immune modulation; predictive analyses suggested possible involvement of PI3K/AKT, MAPK, p53, and HIF-1α/VEGF-related pathways | NMIBC clinical studies in the postoperative setting; network pharmacology/molecular docking analyses | A + E | ( Zhang et al., 2022; Suo et al., 2022; Zhao et al., 2023) |
| Healthy-qi reinforcement | Si Jun Zi Decoction | Deficiency-dominant context | Ginseng Radix et Rhizoma, Atractylodis Macrocephalae Rhizoma, Poria, Glycyrrhizae Radix et Rhizoma | Induced apoptosis and enhanced macrophage-mediated antitumor activity in bladder cancer-bearing mice | **BTT739/T739 mouse models:** macrophage phagocytosis 41% (P<0.05); tumor-inhibition rate 55.5%, maximal tumor-latency 11.3 d, survival 43.3 d; apoptosis 20.11±1.32% vs 9.33±1.62% vs 5.91±1.84% (high-dose vs low-dose vs control, P=0.0001); Fas 12.47±0.51% and FasL 21.30±0.49% in the high-dose group (P=0.0002). | Fas/FasL-mediated apoptosis and macrophage-related antitumor effects | Bladder cancer-bearing mouse models without clearly established NMIBC-specific relevance | C | ( Li et al., 2002, 2005) |
| Healthy-qi reinforcement | Buzhong Yiqi Decoction | Deficiency-dominant; postoperative intravesical-therapy setting | Astragali Radix, Ginseng Radix et Rhizoma, Atractylodis Macrocephalae Rhizoma, Glycyrrhizae Radix et Rhizoma, Angelicae Sinensis Radix, Citri Reticulatae Pericarpium, Cimicifugae Rhizoma, Bupleuri Radix | Reported improvements in immune indices and treatment response, with reductions in selected adverse reactions and VEGF levels | **n=102:** clinical response↑; CD3+, CD4+, CD4+/CD8+↑ and CD8+↓ (P<0.05; exact values NR); overall adverse-event rate, NS (P>0.05).  **n=65 completers:** KPS↑ at 3–6 mo; VEGF↓ at 6–12 mo; urinary WBC↓ at 3 mo (P<0.05); recurrence at 6 mo, 12.12% vs 18.75%, and at 12 mo, 18.18% vs 25.00% (both NS). | T-cell-associated immune modulation and possible inhibition of VEGF-related proangiogenic activity | Post-TURBT NMIBC patients receiving adjunctive intravesical therapy | A | (Wang et al., 2021; Yan et al., 2024) |
| Heat-clearing and dampness-draining | Bazheng San | Dampness–heat; postoperative adjunctive-treatment setting | Plantaginis Semen, Dianthi Herba, Polygoni Avicularis Herba, Talcum, Gardeniae Fructus, Rhei Radix et Rhizoma | Reported reductions in recurrence, treatment-related adverse reactions, and urinary symptoms, with improved quality of life | **n=84:** recurrence, NS within 1 y but ↓ thereafter; adverse events↓ and KPS↑ (P<0.05).  **n=82:** recurrence 14.29% vs 25.00%; adverse events 16.67% vs 37.50%; multidomain QoL↑ (all P<0.05).  **n=50:** urinary irritation/systemic symptoms↓ and clinical response↑ (P<0.05; exact values NR). | Improved tolerance to intravesical therapy; possible inflammatory modulation | Post-TURBT NMIBC clinical studies | A | (Li, 2015; Luo et al., 2015; Pan and Zhang Zhang, 2021) |
| Heat-clearing and detoxifying | Longshe Yangquan Decoction | Dampness–heat–toxin; toxin-related context | Hedyotis diffusa, Scutellaria barbata, Solanum nigrum, Duchesnea indica, and botanical drug(s) | Computational analyses predicted potential effects on proliferation, invasion, and inflammation | NA | Predicted CDK2-, EGFR-, MMP9-, and PTGS2-related mechanisms | Network pharmacology and molecular docking; no direct NMIBC experimental validation in the cited study | E | (Cheng et al., 2022) |
| Blood-activating and stasis-resolving | Didang Decoction | Stasis; stasis–toxin context | Hirudo, Tabanus, Persicae Semen, Rhei Radix et Rhizoma | Reported improvements in quality of life and NMIBC-related symptoms; inhibited bladder cancer cell proliferation in vitro | **Clinical NMIBC study:** post-treatment NMIBC24 scores for urinary symptoms, treatment-related problems, and bowel symptoms were 20.78±18.03 vs 25.32±16.39, 9.85±10.49 vs 14.02±11.89, and 12.12±16.41 vs 17.42±13.09, respectively (Didang Decoction vs control; all between-group P<0.05).  **Mechanistic study:** 21 core targets and 16 pathways were identified; JUN/MMP2 showed differential expression/distribution (P<0.05), and Didang Decoction reduced JUN expression in vitro (P<0.01). | Predictive analyses implicated PI3K/AKT, TNF, MAPK, and HIF-1-related pathways; qPCR supported JUN downregulation | Small NMIBC clinical observation; bladder cancer cell experiments; predictive/network-based analyses | A + C + E | (Shen, 2020; Shen et al., 2020) |
| Blood-activating and stasis-resolving | Guizhi Fuling Wan | Stasis; stasis–toxin context | Cinnamomi Ramulus, Poria, Moutan Cortex, Persicae Semen, Paeoniae Radix Rubra | Inhibited bladder cancer growth and induced cell-cycle arrest/apoptosis in experimental models; relatively low urothelial toxicity was reported in an intravesical-relevant model | **In vitro:** maximum non-cytotoxic dose in normal urothelial cells, 2 mg/mL; selectivity index 8.35 (TSGH 8301) and 19.98 (BFTC 905); S- or G2/M-phase arrest (P<0.01).  **MB49 model:** IC50 ≈0.95 mg/mL; G1 arrest↑ (P<0.01–0.001) and S phase↓ (P<0.05); ROS-positive cells 12.6±0.9% vs 2.1±0.7% (control). **Orthotopic model:** bladder volume 95.1±66 mm³ vs 371.7±335.1/445.1±208 mm³ in untreated/PBS controls; BCG 168.7±123.3 and Mito-C 51.9±38.6 mm³, with no significant difference among the three active-treatment groups. | Cell-cycle arrest/apoptosis involving CHK2/P21- and ATM/P53-related pathways | Bladder cancer cell studies and a mouse model with intravesical relevance | B + C | (Lu et al., 2013, 2016) |
| Healthy-qi reinforcement and mass-resolving | Fuzheng Xiaozheng Decoction | Deficiency with stasis–toxin; postoperative adjunctive-treatment setting | Astragali Radix, Codonopsis Radix, Hedyotis diffusa, Scutellaria barbata, Curcumae Rhizoma, Sparganii Rhizoma, and botanical drug(s) | Reported improvements in selected immune indices and symptoms and reductions in tumor-related and invasion markers | **n=128:** response 89.06% vs 75.00%; adverse events 7.81% vs 25.00%; CD3+, CD4+, CD4+/CD8+↑; CA125, MMP-9, FGF, VEGF, NF-κB p65, ILK and ABCE1↓; PPARγ↑ (all P<0.05). **Experimental study:** proliferation/migration↓ and apoptosis↑; miR-21↓/SMAD7-associated regulation; exact quantitative estimates NR. | GSK3β/β-catenin- and miR-21/SMAD7-related regulation | NMIBC clinical evidence; bladder cancer animal and cell experiments; predictive/network-based analyses | A + C + E | (Zhuang et al., 2023; Yu et al., 2025; Yang et al., 2025) |
| Detoxifying and mass-resolving | Compound Kushen Injection | Heat-toxin/toxin-related; investigated as an adjunct to intravesical or systemic treatment | Sophora flavescens and related components; matrine, oxymatrine, sophoridine, and other alkaloids | Reported improvements in selected immune/inflammatory indices and treatment tolerance; experimental studies reported inhibition of proliferation/invasion and promotion of apoptosis/autophagy | **n=90:** CD3+, CD4+, CD4+/CD8+, IFN-γ and IL-2↑; CD8+, TNF-α, IL-6, CRP, AST/ALT and BUN/Cre↓ vs pirarubicin alone (all P<0.05; exact values NR).  **S637 cells:** proliferation/invasion and VEGF/HIF-1α/SOX-2↓; apoptosis/Caspase-3↑ (dose-dependent, P<0.05; exact values NR).  **MB49 model:** TLR2/TLR4/CD40 positivity and LC3II/LC3I↑; BNIP3/CTSS↓ (exact values NR).  **T24 study:** proliferation/migration↓ and apoptosis↑; quantitative estimates NR. | Autophagy- and macrophage-related regulation; PI3K/AKT/mTOR-, VEGF/HIF-1α-, and MMP-9-related pathways | NMIBC/superficial bladder cancer clinical evidence; broader bladder cancer clinical/meta-analytic evidence; bladder cancer animal/cell studies; bioinformatic and network-pharmacology analyses | A + C + D + E | (Zhang et al., 2017; Cao et al., 2017; Zhang et al., 2022; Zhang et al., 2024; Tian, 2025) |
| Detoxifying and mass-resolving | Huachansu | Toxin-related context | Toad-skin-derived preparation; bufalin and related bufadienolides | Induced apoptosis and inhibited migration/invasion in bladder cancer cells | **T24 cells:** G0/G1 arrest↑ and S-phase fraction/proliferation index altered by cinobufacin (P<0.05; exact values NR). **Bufalin + TRAIL:** sub-G1 cells 23.70% vs 3.14% (bufalin) and 4.10% (TRAIL); viability↓ and apoptosis↑ with combination treatment (exact P values NR). | TRAIL-mediated apoptosis; MMP/tight-junction modulation; possible ERK involvement | Bladder cancer cell/component-based mechanistic studies | C | ( Lüet al., 2010; Kang et al., 2017) |

**Note:** Stratum A, direct NMIBC clinical evidence; Stratum B, NMIBC-relevant translational evidence from postoperative, intravesical, orthotopic, reimplantation, or related models; Stratum C, experimental evidence from general bladder cancer models without specific NMIBC relevance; Stratum D, broader bladder cancer clinical or meta-analytic evidence without separately identifiable NMIBC data; and Stratum E, indirect or hypothesis-generating evidence derived from computational or predictive analyses, including network pharmacology, molecular docking, bioinformatics, and predictive transcriptomics.
