# Supplementary material for: Traditional Chinese medicine in non-muscle-invasive bladder cancer: a scoping review of syndrome characteristics and critical synthesis of pharmacological and mechanistic evidence
Source: Front Pharmacol. 2026 Sep 22;17:1921622. doi: 10.3389/fphar.2026.1921622 (PMC13639432; doi:10.3389/fphar.2026.1921622)
Supplement: Supplementary file 8 [file Table7.docx]

Supplementary Table S 6. Representative plant-derived metabolites and other defined natural products investigated in bladder cancer-related biological processes, with study/model context and evidence-relevance strata.

| **Functional module** | **Natural-product class** | **Representative metabolite or defined natural product** | **Source/related botanical drug or preparation** | **Reported effects** | **Reported mechanisms/pathways** | **Study/model context** | **Evidence-relevance stratum(s)** | **References** |
| --- | --- | --- | --- | --- | --- | --- | --- | --- |
| Tumor-cell proliferation and cell death | Saponin | Compound K | Ginsenoside metabolite | Induced apoptosis in T24 bladder cancer cells | ROS-mediated mitochondrial apoptosis and p38 MAPK activation | T24 bladder cancer cell model | C | (Wang et al., 2013) |
| Tumor-cell proliferation and cell death | Flavonoid | Quercetin | Multiple TCM herbs and plant sources | Inhibited proliferation and colony formation and migration; induced apoptosis | AMPK activation; 4E-BP1/S6K-related regulation; decreased mutant p53 and Survivin | Bladder cancer cell models | C | (Ma et al., 2006; Su et al., 2016) |
| Tumor-cell proliferation and cell death | Flavonoid | Baicalein | *Scutellaria baicalensis* / Huangqin | Induced apoptosis; inhibited proliferation and invasion; slightly reduced orthotopic bladder tumor growth | ROS-dependent caspase activation; p65 NF-κB inhibition; cyclin B1/D1 and MMP-2/9 downregulation | Bladder cancer cell models and an orthotopic bladder tumor mouse model | B + C | (Wu et al., 2013; Choi et al., 2016) |
| Tumor-cell proliferation and cell death | Flavonoid | Baicalin | *Scutellaria baicalensis* / Huangqin | Induced ferroptosis and cell death in bladder cancer models | FTH1-dependent ferroptosis with ROS accumulation and increased intracellular iron | Bladder cancer cell and in vivo tumor models without clearly established NMIBC-specific relevance | C | (Kong et al., 2021) |
| Tumor-cell proliferation and cell death | Flavonoid | Luteolin | Multiple TCM herbs and plant sources | Inhibited proliferation, induced G2/M arrest, and reduced bladder tumor growth | p21 upregulation; mTOR/p-S6 inhibition; Ki-67 reduction | Bladder cancer cell and animal models | C | (Iida et al., 2020) |
| Tumor-cell proliferation and cell death | Flavonoid | Glabridin | Glycyrrhiza glabra | Inhibited proliferation and migration and in vivo tumor growth; induced apoptosis and G2-phase arrest | G2-phase arrest; BIM/BAK1/CYCS-mediated intrinsic apoptosis | Bladder cancer cell and in vivo tumor models | C | (Yang et al., 2023) |
| Tumor-cell proliferation and cell death | Alkaloid | Berberine | *Coptis chinensis*, *Phellodendron chinense*, and botanical drug(s) | Inhibited bladder cancer growth; induced DNA-replication defects, cell-cycle arrest, apoptosis, or senescence | miR-17-5p upregulation; JAK1/STAT3 signaling inhibition | Bladder cancer cell and in vivo tumor models | C | (Xia et al., 2021) |
| Tumor-cell proliferation and cell death | Alkaloid | Febrifugine | Changshan-related herb | Inhibited proliferation and tumor growth; promoted apoptosis | Suppressed DNA synthesis and steroidogenesis/cholesterol-biosynthesis-related processes | Bladder cancer cell and animal models with transcriptomic analysis | C + E | (Chen et al., 2024) |
| Tumor-cell proliferation and cell death | Diterpenoid lactone | Andrographolide | Andrographis paniculata | Inhibited proliferation, migration/invasion, and tumor growth; enhanced apoptosis | NF-κB and PI3K/AKT inhibition; p53-mediated death-receptor upregulation | Bladder cancer cell and xenograft mouse models | C | (Deng et al., 2019; Xuan et al., 2022) |
| Tumor-cell proliferation and cell death | Polysaccharide | Astragalus polysaccharide | *Astragalus membranaceus* / Huangqi | Inhibited proliferation and migration and tumor growth; induced ferroptosis/apoptosis; enhanced cisplatin sensitivity | AMPK/BECN1-xCT/GPX4-mediated ferroptosis; PI3K/AKT/FoxO1 regulation; predicted tumor-microenvironment remodeling | Urothelial/bladder cancer cell and animal models; bioinformatic analyses | C + E | (Tong et al., 2023; Chen et al., 2025) |
| Inflammation and immune microenvironment | Polysaccharide | Polyporus polysaccharide | *Polyporus umbellatus* / Zhuling | Enhanced antitumor immunity; reduced tumor invasiveness and BCG-related side effects | TAM polarization; NF-κB/NLRP3-related signaling; DC/TLR4 activation with increased CD86/CD40 | Bladder cancer BBN rat model and BCG-combination experimental models | B | (Zhang et al., 2015; Jia et al., 2021; Liu et al., 2022) |
| Inflammation and immune microenvironment | Polysaccharide | Lentinan | Lentinus edodes | Inhibited bladder tumor growth and enhanced gemcitabine-related antitumor immunity | Macrophage activation; increased CD4+/CD8+ T cells and IFN-γ/IL-12; reduced Treg/MDSC populations and IL-10/TGF-β | Bladder cancer mouse immunomodulatory model | C | (Sun et al., 2020) |
| Angiogenesis, invasion/migration, and reimplantation | Anthraquinone | Emodin | *Rheum palmatum* and botanical drug(s) | Inhibited bladder cancer cell growth and invasion | Notch1/Jagged1 downregulation; reduced VEGF/VEGFR2 and MMP2 expression | Bladder cancer cell model | C | (Ma et al., 2017) |
| Angiogenesis, invasion/migration, and reimplantation | Polyphenol | EGCG | Green tea | Reduced intravesical tumor-cell implantation and bladder tumor growth | Reduced tumor-cell implantation; transcriptomic and miRNA–mRNA network changes related to inflammatory and oxidation-reduction processes | Intravesical bladder tumor-cell implantation model, bladder cancer cell studies, and transcriptomic/bioinformatic analyses | B + C + E | (Kemberling et al., 2003; Lee et al., 2019) |
| Tumor-cell proliferation and cell death | Phenolic compound | Paeonol | Moutan Cortex | Inhibited proliferation and tumor growth; induced apoptosis | PI3K/AKT signaling inhibition; Bax/Bcl-2/Caspase-3-related apoptosis | Bladder cancer cell and xenograft mouse models | C | (Zhang et al., 2021) |
| Angiogenesis, invasion/migration, and reimplantation | Diterpenoid quinone | Tanshinone IIA | *Salvia miltiorrhiza* / Danshen | Inhibited proliferation, EMT, migration, and invasion | STAT3–CCL2 signaling inhibition; MMP-2/9 suppression and EMT-marker modulation | Bladder cancer cell models | C | (Huang et al., 2017) |
| Angiogenesis, invasion/migration, and reimplantation | Cardiotonic steroid | Bufalin | Huachansu / toad-derived preparation | Inhibited migration/invasion; enhanced TRAIL-mediated apoptosis | TRAIL/death receptor-mediated apoptosis; MMP-2/9 inhibition and tight-junction modulation; possible ERK involvement | T24 bladder cancer cell/component-based studies | C | (Hong et al., 2013; Kang et al., 2017) |
| Angiogenesis, invasion/migration, and reimplantation | β-carboline alkaloid | Harmine | *Peganum harmala* and related sources | Inhibited angiogenesis and xenograft tumor growth; induced apoptosis | VEGF/VEGFR2-mediated angiogenic signaling inhibition; caspase-dependent apoptosis | Bladder cancer xenograft, endothelial-cell, and bladder cancer cell-apoptosis models | C | (Hai-rong et al., 2019) |

**Note:** Evidence-relevance strata reflect study design and proximity to the NMIBC clinical context rather than formal levels of evidence quality. Stratum A, direct NMIBC clinical evidence; Stratum B, NMIBC-relevant translational evidence from postoperative, intravesical, orthotopic, reimplantation, or related models; Stratum C, experimental evidence from general bladder cancer models without specific NMIBC relevance; Stratum D, broader bladder cancer clinical or meta-analytic evidence without separately identifiable NMIBC data; and Stratum E, indirect or hypothesis-generating evidence derived from computational or predictive analyses, including network pharmacology, molecular docking, bioinformatics, and predictive transcriptomics. Multiple strata were assigned when supported by distinct types of evidence. Most evidence summarized in this table derives from bladder cancer experimental models rather than NMIBC-specific clinical studies and should therefore be interpreted as mechanistic or translational evidence rather than direct evidence of clinical efficacy or recurrence prevention in NMIBC. ↑ and ↓ indicate significantly higher and lower values, respectively, compared with the corresponding control group unless otherwise specified; NR, not reported; NS, not statistically significant; wk, week; mo, month; y, year; KPS, Karnofsky Performance Status; QoL, quality of life.
