# Supplementary material for: Traditional Chinese medicine in non-muscle-invasive bladder cancer: a scoping review of syndrome characteristics and critical synthesis of pharmacological and mechanistic evidence
Source: Front Pharmacol. 2026 Sep 22;17:1921622. doi: 10.3389/fphar.2026.1921622 (PMC13639432; doi:10.3389/fphar.2026.1921622)
Supplement: Supplementary file 9 [file Table8.docx]

Supplementary Table S 7. ConPhyMP-informed reporting characteristics of formula- and preparation-level studies.

| **Formula/preparation** | **Reference** | **Study type** | **Composition and dose/ratio reporting** | **Preparation, dosage form and administration** | **Material source/authentication and product traceability** | **Phytochemical characterization/QC** |
| --- | --- | --- | --- | --- | --- | --- |
| Modified Zhibai Dihuang Decoction | (Zhao et al., 2023) | NMIBC clinical | Detailed reporting — Complete 13-herb formula with individual doses reported | Partial reporting — Water decoction, 200 mL/day, divided BID before meals for 8 weeks; detailed decoction parameters not reported | NR | NR |
| Modified Zhibai Dihuang Decoction | (Suo et al., 2022) | NMIBC clinical | Detailed reporting — Complete base formula with individual doses and symptom-based additions reported. | Partial reporting — One water decoction daily, divided BID for 8 weeks; final volume and detailed decoction parameters not reported. | NR | NR |
| Zhibai Dihuang Decoction | (Zhang et al., 2022) | Network pharmacology | Partial reporting — Formula composition defined, but individual botanical-drug doses/ratios not reported. | NA — No physical preparation or administration. | NA | NA |
| Si Jun Zi Decoction | (Li et al., 2005) | Tumor-bearing mouse study | Detailed reporting —Four-botanical-drug formula with a 2:2:2:1 ratio reported | Partial reporting — Extract prepared at 1 or 2 g crude drug/mL; 0.15 mL by gavage, once or twice daily for 14 days; detailed extraction conditions not reported | NR | NR |
| Si Jun Zi Decoction | (Li et al., 2002) | Tumor-bearing mouse study | Partial reporting —Four botanical drugs reported, but individual doses/ratio not reported | Partial reporting — Extract concentrated to 2 g crude drug/mL and stored at 4°C; 0.25 mL by gavage once daily; detailed extraction conditions not reported. | NR | NR |
| Modified Buzhong Yiqi Decoction | (Yan et al., 2024) | NMIBC clinical | Detailed reporting — Complete modified formula with individual botanical-drug doses reported | Partial reporting — Hospital-prepared water decoction, 400 mL/day in two bags, taken BID for 12 weeks; detailed decoction parameters not reported | Partial reporting — Prepared by the hospital TCM preparation room; botanical-drug source, authentication, and batch information not reported | NR |
| Buzhong Yiqi Decoction | (Wang et al., 2021) | NMIBC clinical | Detailed reporting —Complete eight-botanical-drug formula with individual doses reported | Partial reporting — One water decoction daily, divided BID on an empty stomach, continued for 1 year after surgery; detailed decoction parameters not reported | NR | NR |
| Modified Bazheng San | (Pan and Zhang, 2021) | NMIBC clinical | Detailed reporting — Complete base formula with individual doses and symptom-based additions reported | Partial reporting — Water decoction, 200 mL/dose, divided BID after meals; daily for 3 months, then every other day, for 12 months; detailed decoction parameters not reported | NR | NR |
| Modified Bazheng San | (Luo et al., 2015) | NMIBC clinical | Detailed reporting — Complete formula with individual herb doses reported. | Partial reporting — Water decoction, one dose daily, divided BID; treatment continued for >10 months/year; detailed decoction parameters not reported | NR | NR |
| Modified Bazheng San | (Li, 2015) | NMIBC clinical | NR — Exact formula composition and individual doses were not reported in the cited study; the prescription was stated to be clinically modified | Partial reporting — Used alongside intravesical therapy; the overall treatment course was 2 years, but dosage form, preparation method, dose, and administration frequency were not reported in the cited study | NR | NR |
| Longshe Yangquan Decoction (LSYQ) | (Cheng et al., 2022) | Network pharmacology / computational | Partial reporting —Six constituent botanical drugs were specified, but individual doses/ratios were not reported | NA — No physical decoction was prepared or administered | NA — No physical herbal materials requiring source/authentication or batch traceability were used | NA — Constituents were retrieved and screened computationally from TCMSP, HERB, TCM-ID, literature, and SwissADME rather than chemically characterized in an actual decoction |
| Didang Decoction | (Shen et al., 2020) | Network pharmacology | Partial reporting — Four constituent medicines were specified, but individual doses/ratios were not reported | NA — No physical preparation or administration | NA | NA — Targets were derived from TCM and disease databases rather than an experimentally characterized preparation |
| Didang Decoction / Modified Didang Decoction | (Shen, 2020) | NMIBC clinical + network pharmacology + in vitro | Detailed reporting — Clinical modified formula with individual doses and symptom-based additions was reported; the in-vitro formula was specified at a 2:1:3:2 ratio with exact crude-drug amounts | Detailed reporting — Clinical decoction was given daily in two 200-mL doses; the in-vitro preparation included defined soaking, decoction, filtration, concentration, and storage procedures, with cell exposure concentration/time reported | Partial reporting —Clinical botanical drugs were sourced from the hospital pharmacy; experimental materials had specified suppliers and professional authentication, but batch/voucher information was not reported | NR — No chromatographic fingerprint, marker-compound quantification, or other phytochemical quality-control information was reported in the cited study |
| Guizhi Fuling Wan (GFW) extract | (Lu et al., 2013) | In vitro bladder cancer study | Partial reporting — GFW commercial extract was specified, but constituent botanical drugs and formulation ratios were not reported in the cited study | Detailed reporting — Extract was dissolved in ddH₂O, 0.22-μm filtered at 220 mg/mL, and stock concentration was confirmed after lyophilization | Detailed reporting — Commercial extract source (Sun Ten Pharmaceutical) and batch No. 221141 were reported | Detailed reporting — GFW extract was validated by HPLC according to the supplementary experimental procedures |
| Guizhi Fuling Wan (GFW) extract | (Lu et al., 2016) | Orthotopic murine bladder cancer / intravesical study | Partial reporting — GFW extract was specified, but constituent botanical drugs and formulation ratios were not reported in the cited study | Detailed reporting — Extract was purchased, validated and prepared according to a previously described protocol; 100 μL at 180 mg/mL was administered intravesically every other day | Partial reporting — Commercial extract use and a referenced prior preparation protocol were stated, but supplier and batch information were not reported in the cited study | Detailed reporting — Extract validation was explicitly performed according to the previously described HPLC-validated protocol |
| Xiaozheng Decoction | (Zhuang et al., 2023) | Network pharmacology + phytochemical analysis + in vitro study | Detailed reporting —Seven botanical drugs with exact amounts/ratio (30:20:20:20:10:10:10 g) were reported | Detailed reporting —The botanical drugs were soaked for 1 h, decocted with 10-fold water for 30 min, filtered, concentrated to 1.0 g/mL, freeze-dried, and stored at −20°C | Partial reporting —The botanical drugs were purchased from Lei Yun Shang Pharmaceutical Group Co., Ltd., but batch/voucher and botanical authentication were not reported | Detailed reporting — The actual Xiaozheng Decoction was subjected to UPLC-qTOF-MS quality control with detailed chromatographic conditions |
| Xiaozheng Decoction extract | (Yang et al., 2025) | In vitro + xenograft mouse study | Partial reporting — Seven constituent botanical drugs were specified, but individual doses/ratios and the study-specific formulation were not reported | Partial reporting — A laboratory-available extract was used at 50–200 μg/mL in vitro; in-vivo treatment was reported using the extract, but extraction/preparation procedures and complete administration details were not reported | NR —Botanical-drug source, authentication, manufacturer and batch information were not reported | NR — No chromatographic fingerprint, marker-compound quantification, or other phytochemical QC was reported |
| Fuzheng Xiaozheng Decoction | (Yu et al., 2025) | NMIBC clinical | Detailed reporting — Complete eight-herb formula with individual doses and symptom-based additions reported | Partial reporting — Water decoction, concentrated to 400 mL/day, divided BID for 8 weeks; detailed decoction parameters were not reported | NR | NR |
| Compound Kushen Injection | (Cao et al., 2017) | NMIBC clinical | Partial reporting — A standardized commercial preparation was identified, but its constituent composition and formulation ratio were not reported in the cited study | Detailed reporting — Commercial injection, 2 mL intramuscularly BID for 4 weeks; treatment regimen was clearly reported | Detailed reporting — Manufacturer (Shanxi Zhendong Pharmaceutical Co., Ltd.) and Chinese drug approval No. Z14021231 were reported | NR — No chromatographic fingerprint, marker-compound quantification, or other phytochemical quality-control information was reported in the cited study |
| Compound Kushen Injection (CKI) | (Tian, 2025) | Orthotopic bladder cancer mouse study + serum pharmacochemistry/systems biology | Partial reporting — CKI was described as containing Sophora flavescens and Heterosmilax (“Baituling”), but their formulation ratio was not reported | Partial reporting — Commercial injection was administered intraperitoneally once daily at 1, 2, or 4 mL/kg (2 mL/kg in the combination group); treatment duration was not clearly reported | Partial reporting — CKI was supplied by Shanxi Zhendong Pharmaceutical Co., Ltd., but product batch/approval number and botanical authentication were not reported | Partial reporting — UPLC-Q-Exactive-Orbitrap-MS characterized CKI-derived blood-entry constituents and identified 45 active compounds, but no direct batch-specific fingerprint, marker quantification, or QC of the administered CKI product was reported |
| Compound Kushen Injection (CKI) | (Zhang et al., 2022) | Network pharmacology + bioinformatics + in vitro validation | Partial reporting — Two herbal components, *Sophorae Flavescentis Radix* and *Heterosmilacis Rhizoma*, were specified, but their formulation ratio was not reported | Detailed reporting — Commercial CKI was applied to T24 cells at 0–160 μg/mL for 12–48 h; 120 and 160 μg/mL for 24 h were used for downstream validation | Detailed reporting — Manufacturer (Zhendong Pharmaceutical, Shanxi) and batch No. 20171122 were reported | NR — Active compounds were identified computationally from TCMSP/literature; no direct chromatographic fingerprint, marker quantification, or batch-specific phytochemical QC of the experimental CKI was reported |
| Compound Kushen Injection (CKI) | (Zhang et al., 2017) | In vitro bladder cancer study | Partial reporting — Commercial CKI was specified, but constituent botanical drugs and formulation ratio were not reported in the cited study | Detailed reporting — CKI was applied at 0.25, 0.5, and 1.0 mg/mL for 24 h; exposure conditions were clearly reported | Detailed reporting — Manufacturer (Shanxi Zhendong Group Jinjing Pharmaceutical Co., Ltd.) and batch No. 20050701 were reported | NR — No chromatographic fingerprint, marker-compound quantification, or other phytochemical quality-control information was reported in the cited study |
| Huachansu Injection | (Lü et al., 2010) | In vitro bladder cancer study | Partial reporting — A commercial Huachansu Injection was specified, but its constituent composition and formulation ratio were not reported in the cited study | Detailed reporting — Injection was tested at 0.0001–25 mg/mL; combination groups used 2.5 mg/mL Huachansu with MMC at a 1:1 ratio | Partial reporting — Manufacturer (Anhui Jinchan Biochemical Co., Ltd.) and product specification (5 mL/ampoule, 500 mg/mL) were reported, but batch and product-approval information was not reported | NR — No chromatographic fingerprint, marker-compound quantification, or other phytochemical quality-control information was reported in the cited study |

**Note:** Reporting characteristics were descriptively assessed with reference to the ConPhyMP recommendations. “Detailed reporting” indicates that the principal information relevant to the corresponding domain was reported in the cited study, whereas “Partial reporting” indicates that some relevant information was provided but important details remained unavailable. These categories were used for descriptive purposes and do not represent formal ConPhyMP scores. NR, not reported; NA, not applicable; NMIBC, non-muscle-invasive bladder cancer; QC, quality control; HPLC, high-performance liquid chromatography; CKI, Compound Kushen Injection.
