# Supplementary material for: Traditional Chinese medicine in non-muscle-invasive bladder cancer: a scoping review of syndrome characteristics and critical synthesis of pharmacological and mechanistic evidence
Source: Front Pharmacol. 2026 Sep 22;17:1921622. doi: 10.3389/fphar.2026.1921622 (PMC13639432; doi:10.3389/fphar.2026.1921622)
Supplement: Supplementary file 10 [file Table9.docx]

Supplementary Table S 8. Taxonomic and source identification of medicinal materials used in representative TCM formulae and preparations.

| **Part A. Botanical drugs** | | | | | | | | |
| --- | --- | --- | --- | --- | --- | --- | --- | --- |
| **Medicinal material/drug name** | **Formula(s)/preparation(s) in Table S5** | **Source type** | **Accepted scientific name** | **Author citation** | **Family** | **Medicinal part/material** | **Taxonomic source** | **Identification basis/notes** |
| Rehmanniae Radix Praeparata | Zhibai Dihuang Decoction | Botanical | Rehmannia glutinosa | (Gaertn.) Libosch. ex DC. | Orobanchaceae | Prepared root | MPNS; POWO | — |
| Corni Fructus | Zhibai Dihuang Decoction | Botanical | Cornus officinalis | Siebold & Zucc. | Cornaceae | Dried ripe sarcocarp | MPNS; POWO | — |
| Dioscoreae Rhizoma | Zhibai Dihuang Decoction | Botanical | *Dioscorea polystachya* | Turcz. | Dioscoreaceae | Rhizome | MPNS; POWO | — |
| Alismatis Rhizoma | Zhibai Dihuang Decoction | Botanical | *Alisma plantago-aquatica* subsp. *orientale* | (Sam.) Sam. | Alismataceae | Dried tuber | MPNS; POWO | POWO recognizes Alisma plantago-aquatica subsp. orientale (Sam.) Sam. as an accepted infraspecific taxon of A. plantago-aquatica L. |
| Moutan Cortex | Zhibai Dihuang Decoction; Guizhi Fuling Wan | Botanical | *Paeonia* × *suffruticosa* | Andrews | Paeoniaceae | Root bark | MPNS; POWO | — |
| Anemarrhenae Rhizoma | Zhibai Dihuang Decoction | Botanical | *Anemarrhena asphodeloides* | Bunge | Asparagaceae | Rhizome | MPNS; POWO | — |
| Phellodendri Chinensis Cortex | Zhibai Dihuang Decoction | Botanical | Phellodendron chinense | C.K.Schneid. | Rutaceae | Bark | MPNS; POWO | — |
| Ginseng Radix et Rhizoma | Si Jun Zi Decoction; Buzhong Yiqi Decoction | Botanical | Panax ginseng | C.A.Mey. | Araliaceae | Root and rhizome | MPNS; POWO | — |
| Atractylodis Macrocephalae Rhizoma | Si Jun Zi Decoction; Buzhong Yiqi Decoction | Botanical | Atractylodes macrocephala | Koidz. | Asteraceae | Rhizome | MPNS; POWO | — |
| Glycyrrhizae Radix et Rhizoma | Si Jun Zi Decoction; Buzhong Yiqi Decoction | Botanical | *Glycyrrhiza uralensis*; *Glycyrrhiza inflata*; *Glycyrrhiza glabra* | Fisch. ex DC.; Batalin; L. | Fabaceae | Root and rhizome | MPNS; POWO | Three pharmacopoeial botanical sources were retained. |
| Astragali Radix | Buzhong Yiqi Decoction; Fuzheng Xiaozheng Decoction | Botanical | Astragalus mongholicus | Bunge | Fabaceae | Root | MPNS; POWO | — |
| Angelicae Sinensis Radix | Buzhong Yiqi Decoction | Botanical | Angelica sinensis | (Oliv.) Diels | Apiaceae | Root | MPNS; POWO | — |
| Citri Reticulatae Pericarpium | Buzhong Yiqi Decoction | Botanical | *Citrus reticulata* | Blanco | Rutaceae | Dried mature pericarp | MPNS; POWO | — |
| Cimicifugae Rhizoma | Buzhong Yiqi Decoction | Botanical | *Actaea cimicifuga*; *Actaea dahurica*; *Actaea heracleifolia* | L.; (Turcz. ex Fisch. & C.A.Mey.) Franch.; (Kom.) J.Compton | Ranunculaceae | Rhizome | MPNS; POWO | Three pharmacopoeial botanical sources were retained. Former Cimicifuga names were standardized to the currently accepted Actaea names according to POWO. |
| Bupleuri Radix | Buzhong Yiqi Decoction | Botanical | *Bupleurum chinense*; *Bupleurum scorzonerifolium* | DC.; Willd. | Apiaceae | Root | MPNS; POWO | Two pharmacopoeial botanical sources were retained. |
| Plantaginis Semen | Bazheng San | Botanical | *Plantago asiatica*; *Plantago depressa* | L.; Willd. | Plantaginaceae | Seed | Chinese Pharmacopoeia; MPNS; POWO | Two pharmacopoeial botanical sources were retained. |
| Polygoni Avicularis Herba | Bazheng San | Botanical | Polygonum aviculare | L. | Polygonaceae | Aerial part | MPNS; POWO | — |
| Gardeniae Fructus | Bazheng San | Botanical | Gardenia jasminoides | J.Ellis | Rubiaceae | Dried ripe fruit | MPNS; POWO | — |
| Dianthi Herba | Bazheng San | Botanical | *Dianthus chinensis*; *Dianthus superbus* | L.; L. | Caryophyllaceae | Dried aerial parts | Chinese Pharmacopoeia; MPNS; POWO | Two pharmacopoeial botanical sources were retained. |
| Rhei Radix et Rhizoma | Bazheng San; Didang Decoction | Botanical | *Rheum palmatum*; *Rheum tanguticum*; *Rheum officinale* | L.; (Maxim. ex Regel) Balf.; Baill. | Polygonaceae | Root and rhizome | Chinese Pharmacopoeia; MPNS; POWO | Three pharmacopoeial botanical sources were retained. |
| Hedyotis diffusa | Longshe Yangquan Decoction; Fuzheng Xiaozheng Decoction | Botanical | Scleromitrion diffusum | (Willd.) R.J.Wang | Rubiaceae | Not specified in the cited study | MPNS; POWO | *Hedyotis diffusa Willd. is treated by POWO as a synonym of the accepted name Scleromitrion diffusum (Willd.) R.J.Wang.* |
| Scutellaria barbata | Longshe Yangquan Decoction; Fuzheng Xiaozheng Decoction | Botanical | Scutellaria barbata | D.Don | Lamiaceae | Not specified in the cited study | MPNS; POWO | — |
| Solanum nigrum | Longshe Yangquan Decoction | Botanical | Solanum nigrum | L. | Solanaceae | Not specified in the cited study | MPNS; POWO | — |
| Duchesnea indica | Longshe Yangquan Decoction | Botanical | Potentilla indica | (Andrews) Th.Wolf | Rosaceae | Medicinal part not specified in the cited study | MPNS; POWO | *Duchesnea indica (Andrews) Teschem. is treated by POWO as a synonym of the accepted name Potentilla indica (Andrews) Th.Wolf.* |
| Persicae Semen | Didang Decoction; Guizhi Fuling Wan | Botanical | *Prunus persica*; *Prunus davidiana* | (L.) Batsch; (Carrière) Franch. | Rosaceae | Dried mature seed | Chinese Pharmacopoeia; MPNS; POWO | Two pharmacopoeial botanical sources were retained. |
| Cinnamomi Ramulus | Guizhi Fuling Wan | Botanical | Cinnamomum aromaticum | Nees | Lauraceae | Dried tender twig | Chinese Pharmacopoeia; Flora of China; POWO | Chinese Pharmacopoeia records the medicinal source as Cinnamomum cassia Presl. Owing to nomenclatural ambiguity surrounding C. cassia, the source was standardized here to the currently accepted Cinnamomum aromaticum Nees, consistent with POWO and Flora of China. |
| Paeoniae Radix Rubra | Guizhi Fuling Wan | Botanical | *Paeonia lactiflora*; *Paeonia veitchii* | Pall.; Lynch | Paeoniaceae | Dried root | Chinese Pharmacopoeia; MPNS; POWO | Two pharmacopoeial botanical sources were retained. |
| Codonopsis Radix | Fuzheng Xiaozheng Decoction | Botanical | *Codonopsis pilosula*; *Codonopsis pilosula* subsp. *tangshen* | (Franch.) Nannf.; (Oliv.) D.Y.Hong | Campanulaceae | Root | MPNS; POWO | Chinese Pharmacopoeia recognizes Codonopsis pilosula, C. pilosula var. modesta, and C. tangshen as medicinal sources. Under current POWO taxonomy, C. pilosula var. modesta and C. tangshen are treated under accepted infraspecific taxa of C. pilosula. |
| Curcumae Rhizoma | Fuzheng Xiaozheng Decoction | Botanical | *Curcuma phaeocaulis*; *Curcuma kwangsiensis*; *Curcuma aromatica* | Valeton; S.G.Lee & C.F.Liang; Salisb. | Zingiberaceae | Dried rhizome | Chinese Pharmacopoeia; MPNS; POWO | Chinese Pharmacopoeia recognizes Curcuma phaeocaulis, C. kwangsiensis, and C. wenyujin as medicinal sources. POWO treats C. wenyujin Y.H.Chen & C.Ling as a synonym of the accepted name C. aromatica Salisb. |
| Sparganii Rhizoma | Fuzheng Xiaozheng Decoction | Botanical | Sparganium stoloniferum | (Buch.-Ham. ex Graebn.) Buch.-Ham. ex Juz. | Typhaceae | Dried rhizome | Chinese Pharmacopoeia; MPNS; POWO | — |
| Sophora flavescens | Compound Kushen Injection | Botanical | Sophora flavescens | Aiton | Fabaceae | Root | MPNS; POWO | Infraspecific varieties were not specified in the cited studies and were therefore not assigned. |
| **Part B. Non-botanical medicinal materials** | | | | | | | | |
| **Medicinal material/drug name** | **Formula(s)/preparation(s) in Table S5** | **Source type** | **Accepted scientific name** | **Author citation** | **Family** | **Medicinal part/material** | **Taxonomic source** | **Identification basis/notes** |
| Poria | Zhibai Dihuang Decoction; Si Jun Zi Decoction; Guizhi Fuling Wan | Fungal | Wolfiporia cocos | (F.A.Wolf) Ryvarden & Gilb. | Fomitopsidaceae | Dried sclerotium | Chinese Pharmacopoeia; Index Fungorum | Chinese Pharmacopoeia records the medicinal source under the traditional name Poria cocos; Index Fungorum recognizes Wolfiporia cocos (F.A.Wolf) Ryvarden & Gilb. as the current name. |
| Hirudo | Didang Decoction | Animal | Whitmania pigra; Hirudo nipponia; Whitmania acranulata | (Whitman, 1884); Whitman, 1886; (Whitman, 1886) | Hirudinidae | Dried whole body | Chinese Pharmacopoeia; Catalogue of Life/GBIF | Chinese Pharmacopoeia recognizes three medicinal animal sources. Current nomenclature was standardized using Catalogue of Life/GBIF, including the accepted spelling Hirudo nipponia. |
| Tabanus | Didang Decoction | Animal | Not specified | N/A | Tabanidae | Dried whole female insect | Cited study/source | Species-level source was not specified; no retrospective taxonomic assignment was made. |
| Talcum | Bazheng San | Mineral | N/A | N/A | N/A | Talc mineral | Chinese Pharmacopoeia | Non-biological mineral material; taxonomic identification is not applicable. |
| Toad-derived material | Huachansu/Cinobufacin | Animal-derived | Not specified | N/A | Not assigned | Toad-derived preparation | Cited study/product information | Species-level source was not reported in the cited bladder-cancer study; no species was assigned retrospectively. |

**Note:** Accepted scientific names, author citations, and family assignments of botanical drugs were verified using the Medicinal Plant Names Services (MPNS) and Plants of the World Online (POWO), unless otherwise specified. The Chinese Pharmacopoeia was consulted to identify recognized medicinal sources and medicinal parts where applicable. For non-botanical materials, appropriate taxonomic or source databases were used. “Not specified” indicates that species-level identification was not reported in the cited study or source and was therefore not assigned retrospectively. “N/A” indicates that taxonomic identification is not applicable. “—” indicates that no additional identification note was required beyond the sources listed in the table.
